# Safety and efficacy of avapritinib in advanced systemic mastocytosis: the phase 1 EXPLORER trial

**DOI:** 10.1038/s41591-021-01538-9

**Published:** 2021-12-06

**Authors:** Daniel J. DeAngelo, Deepti H. Radia, Tracy I. George, William A. Robinson, Albert T. Quiery, Mark W. Drummond, Prithviraj Bose, Elizabeth O. Hexner, Elliott F. Winton, Hans-Peter Horny, Meera Tugnait, Oleg Schmidt-Kittler, Erica K. Evans, Hui-Min Lin, Brenton G. Mar, Srdan Verstovsek, Michael W. Deininger, Jason Gotlib

**Affiliations:** 1 Department of Medical Oncology, Dana-Farber Cancer Institute, Boston, MA USA; 2 Guy’s & St Thomas’ NHS Foundation Trust, London, UK; 3 ARUP Laboratories, University of Utah, Salt Lake City, UT USA; 4 University of Colorado Anschutz Medical Campus, Aurora, CO USA; 5 University of Michigan, Ann Arbor, MI USA; 6 Beatson Cancer Centre, Glasgow, UK; 7 The University of Texas MD Anderson Cancer Center, Houston, TX USA; 8 Abramson Cancer Center, University of Pennsylvania, Philadelphia, PA USA; 9 Department of Hematology and Medical Oncology, Winship Cancer Institute of Emory University, Atlanta, GA USA; 10 Institute of Pathology, Ludwig-Maximilians University, Munich, Germany; 11 Blueprint Medicines Corporation, Cambridge, MA USA; 12 Versiti Blood Research Institute and Division Hematology and Oncology, Medical College of Wisconsin, Milwaukee, WI USA; 13 Stanford University School of Medicine and Stanford Cancer Institute, Stanford, CA USA

**Keywords:** Myeloproliferative disease, Mast cells, Phase I trials, Myeloproliferative disease

## Abstract

Advanced systemic mastocytosis (AdvSM) is a rare hematologic neoplasm driven by the *KIT* D816V mutation and associated with poor survival. This phase 1 study (NCT02561988) evaluated avapritinib (BLU-285), a selective KIT D816V inhibitor, in patients with AdvSM. The primary endpoints were the maximum tolerated dose, recommended phase 2 dose and safety of avapritinib. Secondary endpoints included overall response rate and changes in measures of mast cell burden. Avapritinib was evaluated at doses of 30–400 mg once daily in 86 patients, 69 with centrally confirmed AdvSM. Maximum tolerated dose was not reached, and 200 mg and 300 mg daily were studied in dose-expansion cohorts. The most frequent adverse events observed were periorbital edema (69%), anemia (55%), diarrhea (45%), thrombocytopenia (44%) and nausea (44%). Intracranial bleeding occurred in 13% overall, but in only 1% of patients without severe thrombocytopenia (platelets <50 × 10^9^/l). In 53 response-evaluable patients, the overall response rate was 75%. The complete remission rate was 36%. Avapritinib elicited ≥50% reductions in marrow mast cells and serum tryptase in 92% and 99% of patients, respectively. Avapritinib induced deep and durable responses, including molecular remission of *KIT* D816V in patients with AdvSM, and was well tolerated at the recommended phase 2 dose of 200 mg daily.

## Abstract (web summary)

In a phase 1 trial of patients with advanced systemic mastocytosis, avapritinib, a selective KIT inhibitor, was generally well tolerated, elicited durable clinical responses and led to reductions in mast cell disease burden.

## Main

Systemic mastocytosis (SM) is a rare hematologic neoplasm characterized by accumulation of neoplastic mast cells in the bone marrow (BM), skin and other organs^1–4^. Mast cell mediator release and SM-related organ damage (known as C-findings) lead to severe, debilitating symptoms^5,6^. AdvSM comprises three subtypes: aggressive SM (ASM), SM with an associated hematologic neoplasm (SM-AHN), which accounts for ~60–70% of advanced disease^7–10^, and mast cell leukemia (MCL)^6^. Median overall survival (OS) in patients with AdvSM is ≤3.5 years, usually due to organ damage and/or progression of the associated hematologic neoplasm (AHN)^1,5,6,11–14^.

SM is driven by the *KIT* p.Asp816Val (D816V) mutation in approximately 95% of cases^14–16^, yet until recently, therapies designed to specifically target *KIT* D816V were unavailable^6,12,17^. Imatinib lacks activity against KIT D816V^18–20^ and is approved in the United States for the rare patients who have ASM without *KIT* D816V mutation or unknown *KIT* mutation status^21,22^. The multikinase inhibitor midostaurin is approved for AdvSM; however, few patients achieve complete remission, and gastrointestinal adverse events (AEs) are common^7,8,11,23,24^. Safe and effective therapies for patients with AdvSM remain an area of unmet need.

Avapritinib (BLU-285, Blueprint Medicines Corporation) is a new, selective type 1 KIT inhibitor with high potency for KIT D816V^12,24–26^. The US Food and Drug Administration (FDA) approved avapritinib in June 2021 for adult patients with AdvSM, including patients with ASM, SM-AHN and MCL. It was also recently approved in the United States for the treatment of adults with unresectable or metastatic gastrointestinal stromal tumor (GIST) harboring platelet-derived growth factor receptor alpha (*PDGFRA*) exon 18 mutations at a recommended starting dose of 300 mg once daily (QD), based on dose-escalation and dose-expansion trials that evaluated efficacy and safety in this setting^27,28^. We describe the results of a phase 1 international, multicenter, open-label study (EXPLORER, NCT02561988) evaluating the safety, pharmacokinetics (PK), efficacy and patient-reported outcomes (PROs) of avapritinib in adult patients with AdvSM and other myeloid malignancies.

## Results

### Patients

**Fig. 1:**
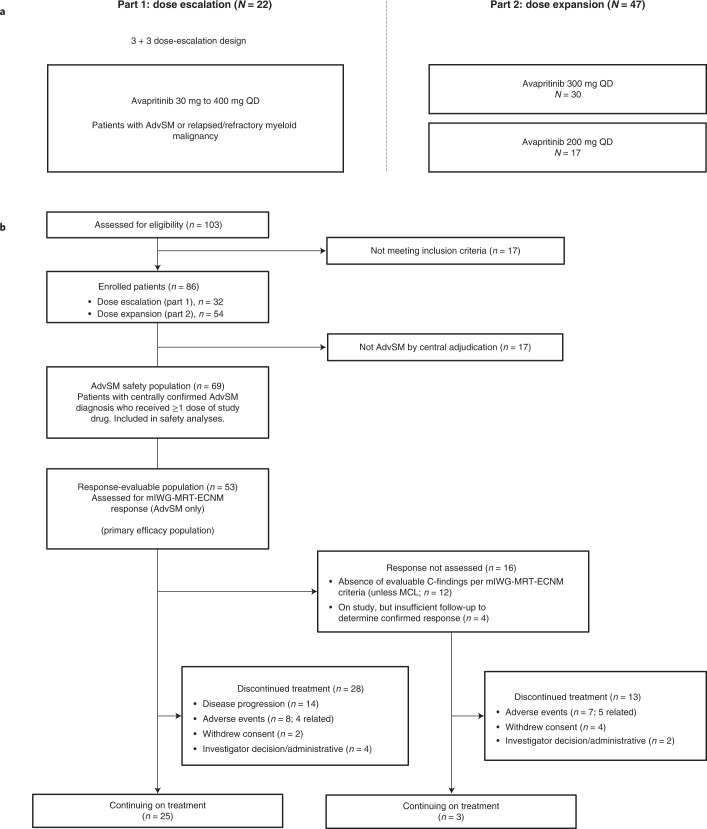
Study design and patient disposition. **a**, Study design showing patients with confirmed AdvSM. **b**, Patient disposition. AdvSM, advanced systemic mastocytosis; mIWG-MRT-ECNM, modified International Working Group-Myeloproliferative Neoplasms Research and Treatment and European Competence Network on Mastocytosis; MCL, mast cell leukemia; QD, once daily.

Between 10 March 2016 and 18 March 2020, 86 patients (dose escalation, *n* = 32; dose expansion, *n* = 54) were enrolled at ten sites in the United States and the United Kingdom (Fig. 1).

Diagnoses were centrally confirmed retrospectively by review of pathology and adjudication of C-findings using World Health Organization (WHO) criteria (Methods). Of note, previously undiagnosed AHN was identified by central pathology in 41% of patients with a local diagnosis of ASM, resulting in reclassification as SM-AHN for analysis. Overall, 69 of the 86 enrolled patients had confirmed AdvSM, while the remaining patients were adjudicated to have diagnoses of indolent SM (*n* = 14), smoldering SM (*n* = 2) or chronic myelomonocytic leukemia (*n* = 1). Modified International Working Group-Myeloproliferative Neoplasm Research and Treatment-European Competence Network on Mastocytosis (mIWG-MRT-ECNM) response outcomes are reported here for patients with a centrally adjudicated diagnosis of AdvSM and at least one evaluable mIWG-MRT-ECNM C-finding (the response-evaluable population), while safety is reported in all treated patients and for those with AdvSM.

At baseline, measures of mast cell disease burden (BM mast cell burden, serum tryptase level) were similar in the safety, AdvSM safety and response-evaluable populations (Table 1). The most common evaluable C-findings per mIWG-MRT-ECNM criteria at baseline were marked splenomegaly (53%), thrombocytopenia (36%), elevated alkaline phosphatase (34%), anemia (32%) and ascites (19%; Supplementary Table 1).

**Table 1:** Patient demographics and baseline disease characteristics^a^

	All enrolled patients (*n* = 86)	AdvSM safety population (*n* = 69)	mIWG-MRT-ECNM response-evaluable population (*n* = 53)
Age, median years (range)	64 (34–83)	67 (34–83)	65 (34–83)
Sex, *n* (%)			
Female	40 (47)	28 (41)	23 (43)
Male	46 (53)	41 (59)	30 (57)
ECOG performance status, *n* (%)			
0–1	64 (74)	48 (70)	36 (68)
2–3	22 (26)	21 (30)	17 (32)
SM subtype per central assessment, *n* (%)			
AdvSM	69 (80)	69 (100)	53 (100)
ASM	8 (9)	8 (12)	3 (6)
SM-AHN	48 (56)	48 (70)	37 (70)
MCL	13 (15)	13 (19)	13 (25)
ISM or SSM	16 (19)	0	0
Other	1 (1)	0	0
*KIT* mutation status per central assay, *n* (%)*			
p.Asp816Val (D816V) positive	78 (91)	64 (93)	51 (96)
p.Asp816Tyr (D816Y) positive	2 (2)	2 (3)	2 (4)
* KIT* mutation negative	6 (7)	3 (4)	0
*S/A/R* mutation per central assay, *n* (%)			
Positive	39 (45)	36 (52)	27 (51)
Negative	47 (55)	33 (48)	26 (49)
Prior antineoplastic therapy, *n* (%)			
Any	51 (59)	41 (59)	32 (60)
Midostaurin	25 (29)	23 (33)	17 (32)
Cladribine	14 (16)	10 (14)	7 (13)
Imatinib	8 (9)	5 (7)	3 (6)
Interferon	4 (5)	4 (6)	3 (6)
BM mast cell burden by central pathology review, median percentage (range)^b^	30 (5–95)	40 (5–95)	50 (5–95)
Serum tryptase level per central assay, median ng ml^−1^ (range)	158 (12–1,414)	173 (12–1,414)	182 (21–765)
Spleen volume, median ml (range)^c^	762 (130–2,300)	994 (149–2,300)	1101 (258–2,300)
*KIT* D816V variant allele fraction, median percentage (range)^d^	9 (0–81)	14 (0–81)	17 (0–81)
Skin involvement by mastocytosis, *n* (%)	32 (37)	22 (32)	19 (36)

^a^Percentages may not total 100 because of rounding; *KIT D816V mutation status assessed by central ddPCR assay; all coding regions of *KIT* D816Y were assessed by central Illumina Trusight Myeloid panel.

^b^Median values (%) in *n* = 71, *n* = 64 and *n* = 43 patients in all enrolled patients, the AdvSM safety population and mIWG-MRT-ECNM response-evaluable population, respectively.

^c^Spleen volume assessed by central radiology review of MRI or CT imaging (*n* = 78, *n* = 66 and *n* = 47 for all enrolled patients, the AdvSM safety population and mIWG-MRT-ECNM response-evaluable population, respectively).

^d^Mutated allele fraction (%) assessed using validated ddPCR by central assay with a limit of detection of 0.17%.

AdvSM, advanced systemic mastocytosis; ASM, aggressive systemic mastocytosis; ddPCR, droplet digital polymerase chain reaction; ECOG, Eastern Cooperative Oncology Group; ISM, indolent systemic mastocytosis; MCL, mast cell leukemia; mIWG-MRT-ECNM, modified International Working Group-Myeloproliferative Neoplasms Research and Treatment and European Competence Network on Mastocytosis; *S/A/R, SRSF2*, *ASXL1*, or *RUNX1*; SM, systemic mastocytosis; SM-AHN, systemic mastocytosis with an associated hematologic neoplasm; SSM, smoldering systemic mastocytosis.

Forty-one of 69 patients with AdvSM (59%) had received prior antineoplastic therapy, including 23 (33%) previously treated with midostaurin and 10 (14%) with cladribine (Supplementary Table 2). Thirty-six patients with AdvSM (52%) had at least one mutation in the *SRSF2*, *ASXL1* or *RUNX1* (*S/A/R*) genes, which are associated with poor disease prognosis^29^.

**Table 2:** Adverse events

	All patients (*n* = 86)		AdvSM safety population (*n* = 69)	
	Any grade	Grade ≥ 3	Any grade	Grade ≥ 3
**Non-hematologic AEs in** ≥**15% patients,** ***n*** **(%)**				
Periorbital edema	59 (69)	2 (2)	45 (65)	1 (1)
Diarrhea	39 (45)	1 (1)	30 (43)	0
Nausea	38 (44)	3 (3)	29 (42)	3 (4)
Fatigue	35 (41)	8 (9)	24 (35)	7 (10)
Peripheral edema	34 (40)	0	31 (45)	0
Vomiting	31 (36)	4 (5)	22 (32)	3 (4)
Arthralgia	24 (28)	3 (3)	18 (26)	2 (3)
Hair color changes	22 (26)	1 (1)	13 (19)	1 (1)
Decreased appetite	20 (23)	1 (1)	15 (22)	1 (1)
Memory impairment	20 (23)	0	14 (20)	0
Dizziness	19 (22)	2 (2)	14 (20)	1 (1)
Constipation	19 (22)	1 (1)	14 (20)	1 (1)
Abdominal pain	19 (22)	1 (1)	13 (19)	0
Headache	18 (21)	1 (1)	11 (16)	1 (1)
Hypokalemia	18 (21)	3 (3)	14 (20)	2 (3)
Upper respiratory tract infection	16 (19)	1 (1)	12 (17)	1 (1)
Dysgeusia	16 (19)	0	10 (14)	0
Pruritus	16 (19)	0	11 (16)	0
Dyspnea	15 (17)	0	12 (17)	0
Epistaxis	14 (16)	0	13 (19)	0
Pain in extremity	14 (16)	0	12 (17)	0
Cough	14 (16)	0	10 (14)	0
Cognitive disorder	13 (15)	1 (1)	8 (12)	1 (1)
Hypoesthesia	13 (15)	0	8 (12)	0
Insomnia	13 (15)	1 (1)	10 (14)	2 (3)
Urinary tract infection	13 (15)	1 (1)	9 (13)	1 (1)
**Hematologic AEs ≥15% patients,** ***n*** **(%)**				
Anemia	47 (55)	26 (30)	40 (58)	25 (36)
Thrombocytopenia	33 (44)	29 (34)	35 (51)	28 (41)
Neutropenia	17 (20)	13 (15)	15 (22)	13 (19)

AdvSM, advanced systemic mastocytosis; AEs, adverse events.

### Maximum tolerated dose and recommended phase 2 dose

**Extended Data Fig. 1:**
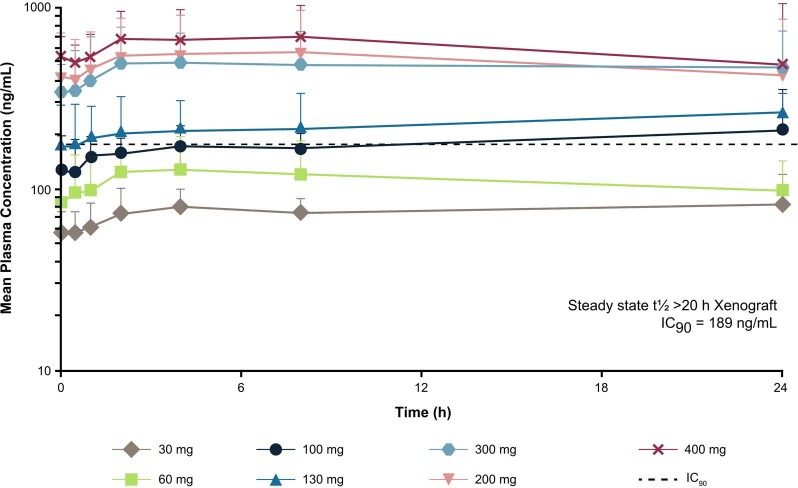
Steady State Mean Plasma Concentration–Time Profiles of Avapritinib in Patients with Systemic Mastocytosis. The figure shows the mean steady state plasma concentrations of avapritinib assessed over time (Cycle 1 Day 15) in patients who received different doses of avapritinib. At most time points, avapritinib plasma concentrations at doses ≥130 mg exceeded the IC_90_ of 189 ng/mL for inhibition of *KIT* D816V measured by immunoblot in a xenograft model. Data are presented as mean values + standard deviation. The number of patients who received each dose are as follows: 30 mg, n = 3; 60 mg, n = 5; 100 mg, n = 3; 130 mg, n = 3; 200 mg, n = 21; 300 mg, n = 43; 400 mg, n = 7. h, hours; IC_90_, 90% inhibitory concentration; t_1/2_ terminal half-life.

Part 1 studied doses of 30, 60, 100, 130, 200, 300 and 400 mg QD, with doses of 200 mg and higher achieving exposures consistently above, and doses of 100 mg and 130 mg partly above, the xenograft 90% inhibitory concentration (IC_90_) for KIT D816V inhibition at steady state (Results and Extended Data Fig. 1). No maximum tolerated dose (MTD) was determined. During part 1, one patient experienced dose-limiting toxicity (DLT) of grade 4 vomiting at 400 mg daily. A recommended phase 2 dose (RP2D) of 300 mg QD was initially selected for use in part 2; however, dose reduction to 200 mg QD was typical, most commonly for cytopenias. Importantly, 200 mg QD had similar exposure and time to response, so a second expansion cohort with a 200 mg QD starting dose was added by protocol amendment. In total, 15 patients initiated treatment at <200 mg QD, 21 patients initiated at 200 mg QD, 43 patients initiated at 300 mg QD and 7 patients initiated at 400 mg QD.

### Safety

**Extended Data Fig. 2:**
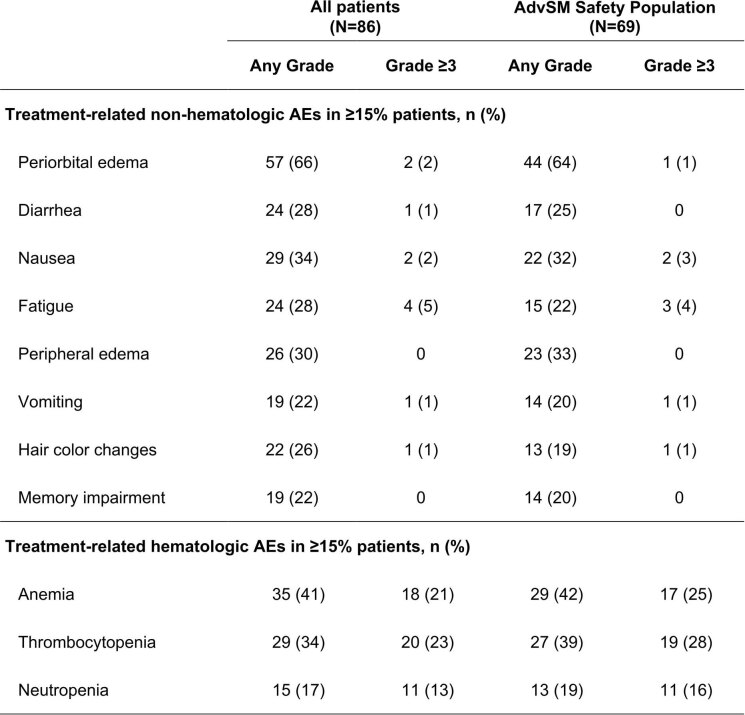
Treatment-Related Adverse Events Reported in ≥15% of Patients. AEs, adverse events.

At data cutoff, 35 of 69 patients with AdvSM (51%) had discontinued treatment: 14 (20%) due to disease progression, 7 (10%) due to related AEs, 6 (9%) due to unrelated AEs, 3 (4%) due to consent withdrawal and 5 (7%) due to investigator decision or administrative causes. The most frequent AEs are presented in Table 2, for the safety and AdvSM safety populations, and treatment-related AEs as determined by the investigator are presented in Extended Data Fig. 2.

**Table 3:** Overall response rates by centrally adjudicated mIWG-MRT-ECNM response criteria

Best confirmed response by mIWG-MRT-ECNM criteria, *n* (%)	By AdvSM subtype				All AdvSM, by midostaurin history		All AdvSM, by prior therapy history	
	All AdvSM (*n* = 53)	ASM (*n* = 3)	SM-AHN (*n* = 37)	MCL (*n* = 13)	Prior midostaurin exposure (*n* = 17)	Midostaurin naïve (*n* = 36)	Any prior therapy (*n* = 32)	No prior therapy (*n* = 21)
ORR (CR + CRh + PR + CI), *n* (%)	40 (75)	3 (100)	28 (76)	9 (69)	10 (59)	30 (83)	22 (69)	18 (86)
95% CI	62–86	29–100	59–88	39–91	33–82	67–94	50–84	64–97
**Best response**								
CR or CRh	19 (36)	2 (67)	14 (38)	3 (23)	3 (18)	16 (44)	9 (28)	10 (48)
CR	8 (15)	0	5 (14)	3 (23)	2 (12)	6 (17)	4 (13)	4 (19)
CRh	11 (21)	2 (67)	9 (24)	0	1 (6)	10 (28)	5 (16)	6 (29)
PR	18 (34)	1 (33)	13 (35)	4 (31)	6 (35)	12 (33)	11 (34)	7 (33)
CI	3 (6)	0	1 (3)	2 (15)	1 (6)	2 (6)	2 (6)	1 (5)
SD	12 (23)	0	8 (22)	4 (31)	6 (35)	6 (17)	9 (28)	3 (14)
PD	0	0	0	0	0	0	0	0
NE	1 (2)	0	1 (3)	0	1 (6)	0	1 (3)	0

AdvSM, advanced systemic mastocytosis; ASM, aggressive systemic mastocytosis; CI, clinical improvement; CR, complete remission; CRh, complete remission with partial recovery of peripheral blood counts; mIWG_MRT-ECNM, modified International Working Group-Myeloproliferative Neoplasms Research and Treatment and European Competence Network on Mastocytosis; MCL, mast cell leukemia; NE, not evaluable; ORR, overall response rate; PD, progressive disease; PR, partial remission; SD, stable disease; SM-AHN, systemic mastocytosis with an associated hematologic neoplasm.

In patients with AdvSM (*n* = 69), the most frequent non-hematologic AEs of any grade were periorbital edema (65%), peripheral edema (45%), diarrhea (43%), nausea (42%), fatigue (35%) and vomiting (32%). The most frequent non-hematologic AEs of grade 3 or 4 were fatigue (10%), nausea and vomiting (both 4%), and arthralgia and hypokalemia (both 3%). The incidence of non-hematologic AEs was comparable between all patients and those with confirmed AdvSM.

In patients with AdvSM, the most frequent hematologic AEs (any grade and grade 3 or 4) were anemia (58% and 36%), thrombocytopenia (51% and 41%) and neutropenia (22% and 19%). Patients with baseline cytopenias were more likely to have cytopenia AEs on study. The risk of ≥grade 3 thrombocytopenia was 20% in the absence of baseline thrombocytopenia, but 70% where thrombocytopenia was present at baseline. Neutropenia of ≥grade 3 occurred in 25% without, and 63% with baseline neutropenia. For ≥grade 3 leukopenia, the risk ratios were 67% versus 19%, with or without any baseline leukopenia, respectively.

Hair color changes, typically grade 1, occurred in 13 patients (19%), and grade 1 skin hypopigmentation was reported in two patients (3%), possibly consistent with inhibition of wild-type KIT. There was one case of anaphylactic reaction (not considered treatment related).

**Extended Data Fig. 3:**
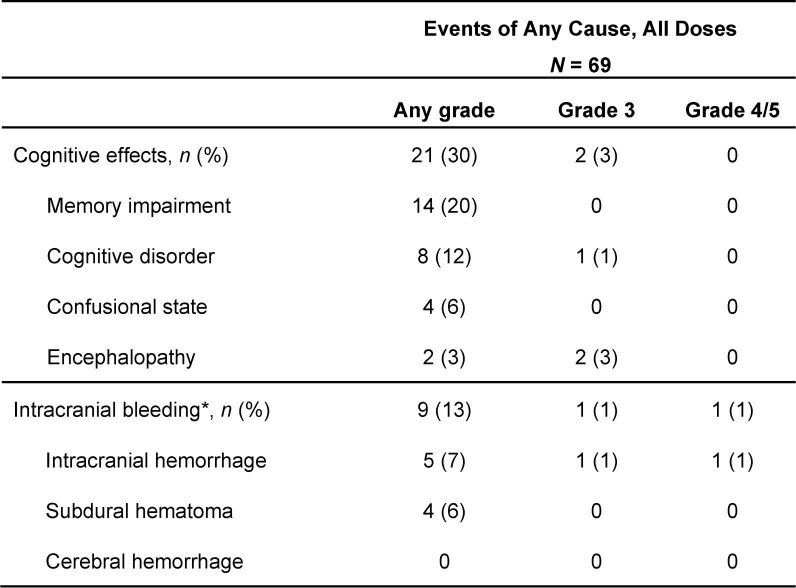
Summary of Cognitive Effects and Intracranial Bleeding Events by Grade Occurring at Any Dose (AdvSM Safety Population). *Two events were associated with head trauma and one occurred in the context of progressive mast cell leukemia. AdvSM, advanced systemic mastocytosis.

Cognitive effects (memory impairment, cognitive disorder, confusional state and encephalopathy) occurred in 21 patients (30%). These events were mostly grade 1 (20%) or grade 2 (7%; Extended Data Fig. 3). Grade 3 cognitive effects occurred in two patients (3%), each in the setting of concomitant benzodiazepine and/or opioid treatment. Cognitive effects were less frequent with starting doses of ≤200 mg and rarely led to treatment discontinuation (two patients; 3%).

**Extended Data Fig. 4:**
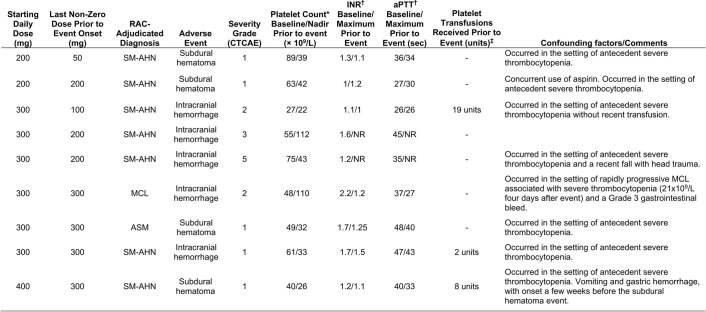
Antecedent Dose, Platelet Count, Prothrombin International Normalized Ratio (INR), Activated Partial Thromboplastin Time (aPTT) and Platelet Transfusions in Patients Who had Intracranial Bleeding Events (AdvSM Safety Population). *Nadir platelet count presented as value during screening/nadir value within 56 days prior to event onset. ^†^Baseline INR and aPTT presented as values during screening/maximum values within 56 days prior to event onset. ^‡^Total number of units of platelets given by transfusion, after first dose of avapritinib and prior to the onset of a first intracranial bleeding event. AdvSM, advanced systemic mastocytosis; aPTT, activated partial thromboplastin time; ASM, aggressive systemic mastocytosis; CTCAE, Common Terminology Criteria for Adverse Events; INR, prothrombin international normalized ratio; MCL, mast cell leukemia; NR, not recorded; RAC, Response Adjudication Committee; SM-AHN, systemic mastocytosis with an associated hematologic neoplasm.

Intracranial bleeding (ICB) was reported in nine patients with AdvSM (13%; Extended Data Fig. 3). Five of the events were asymptomatic (grade 1) and identified by prespecified imaging per protocol, two were grade 2, one was grade 3 and one was grade 5 (which was associated with head trauma). Notably, seven of the events were associated with antecedent severe thrombocytopenia (platelet count <50 × 10^9^/l), and one additional ICB event occurred in the context of rapidly progressive MCL associated with severe thrombocytopenia. Only one (1%) patient experienced ICB in the absence of prior severe thrombocytopenia. Avapritinib dose, platelet count, coagulation studies and platelet transfusions for patients who had ICB are reported in Extended Data Fig. 4. Due to the increased incidence of ICB in association with antecedent severe thrombocytopenia, the protocol was amended to exclude the enrollment of new patients with severe thrombocytopenia (<50 × 10^9^/l), to increase monitoring of the platelet count, and to provide guidance for pausing avapritinib and providing platelet transfusion support for severe thrombocytopenia.

**Extended Data Fig. 5:**
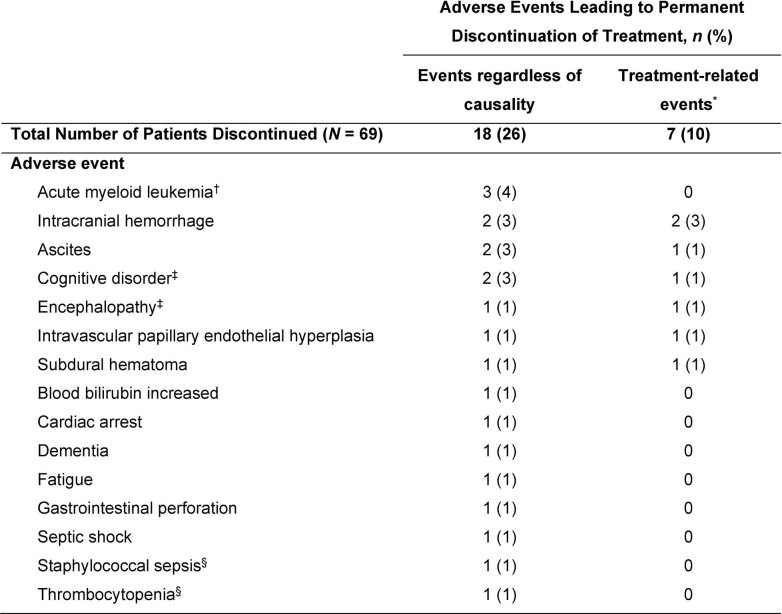
Adverse Events Leading to Treatment Discontinuation (AdvSM Safety Population, *N* = 69). *Events determined to be probably or possibly related to the study medication. ^†^Acute myeloid leukemia is a progressive disease event that was also reported as an AE in these 3 cases. ^‡^Cognitive disorder and encephalopathy occurred in the same patient. ^§^Staphylococcal sepsis and thrombocytopenia occurred in the same patient. AdvSM, advanced systemic mastocytosis.

Fifty (72%) patients with AdvSM experienced at least one dose reduction due to AEs (most commonly for cytopenias), with a median time to first dose reduction of 8 weeks (range, 0–109 weeks). The median daily dose was 164 mg QD (range, 30–317 mg) for all patients, which was similar for patients who started at 200 mg QD. Treatment-related AEs associated with treatment discontinuation are shown in Extended Data Fig. 5. There were six deaths due to AEs (acute myeloid leukemia, gastric hemorrhage, ICB (considered treatment related; occurring in the setting of antecedent severe thrombocytopenia and a recent fall with head trauma), cardiac arrest, staphylococcal sepsis and septic shock).

### Efficacy in patients with AdvSM

Fifty-three patients with AdvSM with baseline measurable C-finding(s) were evaluable for adjudicated response assessment per mIWG-MRT-ECNM criteria; this response-evaluable population comprised 3 patients with ASM, 37 patients with SM-AHN and 13 patients with MCL. Among these patients, overall response rate (ORR) was 75% (40/53; 95% confidence interval (CI), 62–86%), with 19 (36%) exhibiting complete remission with full hematologic recovery (CR) or complete remission with partial hematologic recovery (CRh; Table 3). In addition, 18 (34%) achieved a partial remission (PR), and three (6%) demonstrated clinical improvement. The ORR was 83% (30/36; 95% CI, 67–94%) in midostaurin-naïve patients, and 59% (10/17; 95% CI, 33–82%) in patients with prior midostaurin exposure. The CR/CRh rate was 44% (16/36) in midostaurin-naïve patients and 18% (3/17) in those with prior midostaurin exposure. The CR and CRh rates were 17% (6/36) and 28% (10/36) in midostaurin-naïve patients, and 12% (2/17) and 6% (1/17) in patients with prior midostaurin exposure, respectively. The ORRs in patients with co-mutations in poor-prognosis *S/A/R* genes were similar to those without (74% versus 77%, respectively).

**Extended Data Fig. 6:**
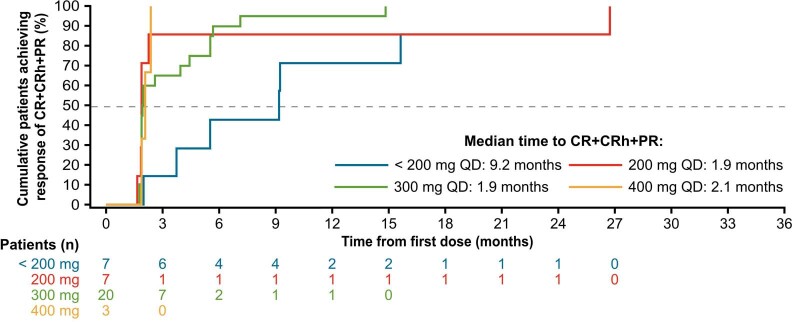
Impact of Starting Dose on Time to Response (CR/CRh/PR) per Modified IWG-MRT-ECNM Criteria Among Responders in the Response-Evaluable Population. CR, complete remission; CRh, CR with partial recovery of peripheral blood counts; IWG-MRT-ECNM, International Working Group-Myeloproliferative Neoplasms Research and Treatment and European Competence Network on Mastocytosis; QD, once daily; PR, partial remission.

The median time to achieve PR or better (CR/CRh/PR) was 2 months (range, 2–27 months), and 9 months to achieve CR/CRh (Supplementary Fig. 1). There was a shorter median time to PR or better at starting doses of 200, 300 or 400 mg (each 2 months) compared with the median time to PR or better at starting doses of <200 mg (9 months; Extended Data Fig. 6). Median duration of response (DOR) was 38 months (95% CI, 22 months–not estimable). Estimated 12-month and 24-month DOR rates were 84% (95% CI, 72–96%) and 67% (95% CI, 49–‍84%), respectively.

**Fig. 2:**
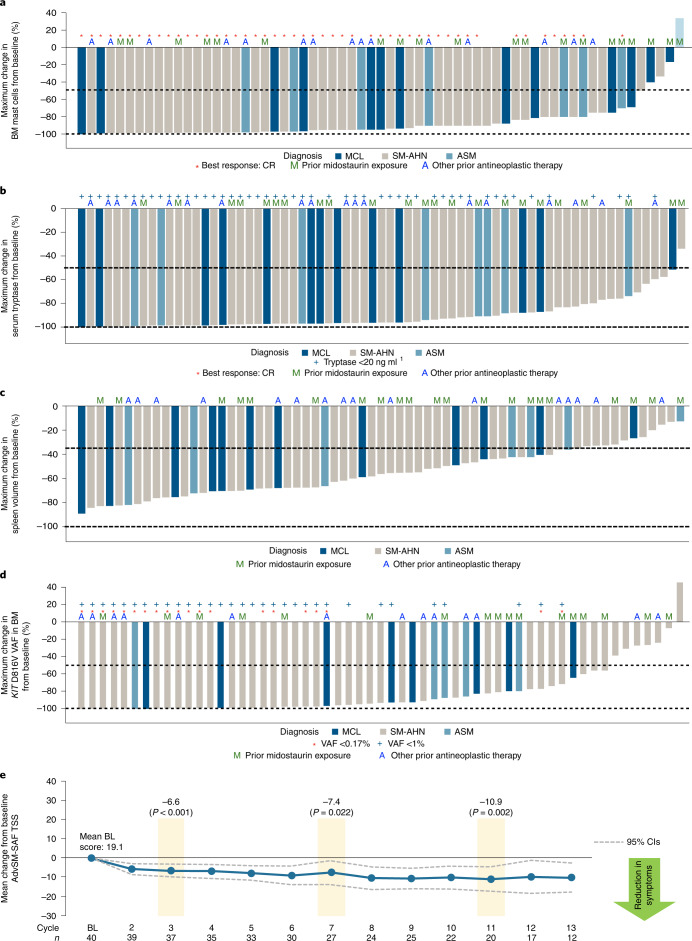
Clinicopathologic measures of response and change in patient-reported measures of symptom burden by AdvSM-SAF score. **a**, BM mast cell burden. **b**, Serum tryptase level. **c**, Spleen volume. **d**, *KIT* D816V VAF in BM, assessed by central ddPCR assay. **e**, Change from baseline in systemic mastocytosis symptom burden, evaluated by AdvSM-SAF TSS. For the change in TSS by AdvSM-SAF, a two-sided paired *t*-test was performed for the change from baseline at C3 (*P* < 0.001), C7 (*P* = 0.022) and C11 (*P* = 0.002). BL, baseline; CR, complete remission; VAF, variant allele fraction. AdvSM-SAF, advanced systemic mastocytosis symptom assessment form; ASM, aggressive systemic mastocytosis; BM, bone marrow; C, cycle; CIs, confidence intervals; ddPCR, droplet digital polymerase chain reaction; MCL, mast cell leukemia; SM-AHN, systemic mastocytosis with an associated hematologic neoplasm; TSS, total symptom score.

**Extended Data Fig. 7:**
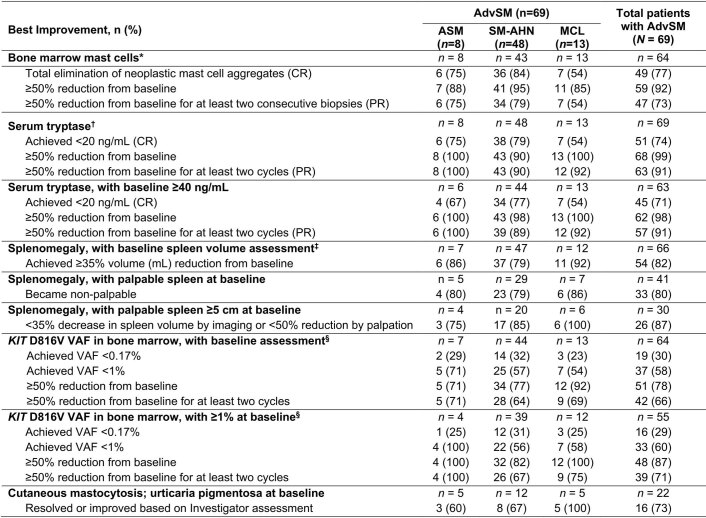
Summary of Clinical Improvement in Measures of Mast Cell Burden (AdvSM Safety Population). *n values indicate number of patients with neoplastic mast cell aggregates at baseline. ^†^*n* values indicate number of patients with baseline serum tryptase assessment. ^‡^Images by CT or MRI centrally assessed by independent radiologist. ^§^Based on central laboratory testing. AdvSM, advanced systemic mastocytosis; ASM, aggressive systemic mastocytosis; CR, complete remission; CT, computed tomography; MCL, mast cell leukemia; MRI, magnetic resonance imaging; PR, partial remission; SM-AHN, systemic mastocytosis with an associated hematologic neoplasm; VAF, variant allele fraction.

Improvements in measures of mast cell burden in patients with AdvSM are presented in Fig. 2a–d and Extended Data Fig. 7. BM mast cells decreased by ≥50% in 92% of patients and mast cell aggregates were eliminated in 77% of patients. Serum tryptase was reduced by ≥50% in 99% of patients and reduced to <20 ng ml^−1^ in 74% of patients. Spleen volume was reduced by ≥35% in 82% of 66 patients with a baseline spleen volume assessment. The *KIT* D816V variant allele fraction (VAF) in BM was reduced from baseline by ≥50% in 80% of patients and became undetectable in 30% of patients. Clinical improvements in C-findings per mIWG-MRT-ECNM criteria observed in response-evaluable patients are presented in Supplementary Table 1.

The depth of clinical response was generally correlated with the elimination of measurable *KIT* D816V allele burden in the BM, with a limit of detection of 0.17% by droplet digital polymerase chain reaction (ddPCR; Supplementary Fig. 2). A post hoc analysis according to best mIWG-MRT-ECNM response showed that complete elimination of measurable *KIT* D816V VAF occurred in 63% patients with a CR (*n* = 8), in 50% patients with a CRh (*n* = 8), in 23% patients with a PR (*n* = 13) and in only 15% of patients with a clinical improvement (*n* = 2) or stable disease (*n* = 11).

**Fig. 3:**
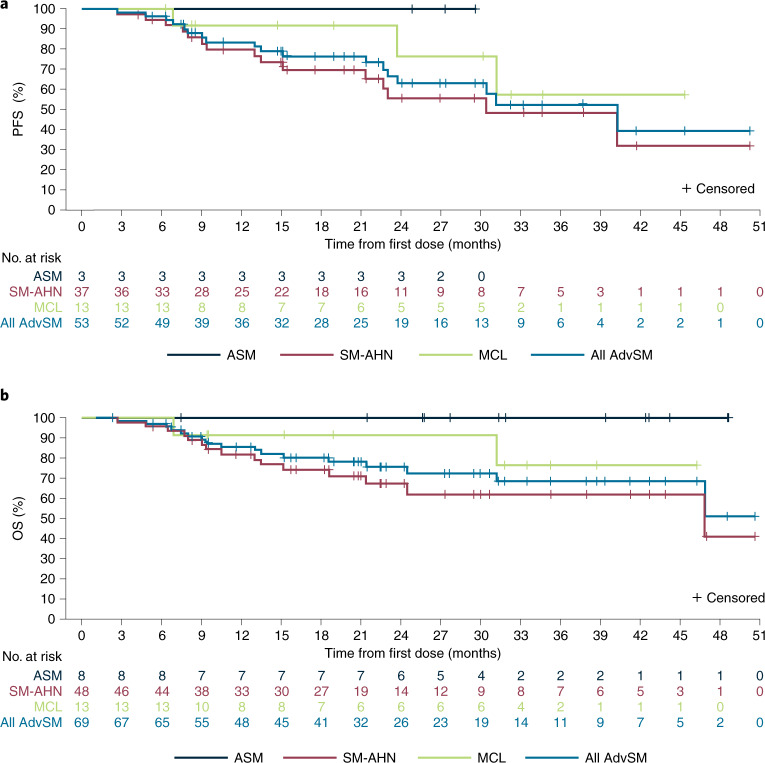
Kaplan–Meier estimates of overall survival and progression-free survival. **a**, PFS in the response-evaluable population. **b**, OS in the AdvSM safety population. AdvSM, advanced systemic mastocytosis; ASM, aggressive systemic mastocytosis; MCL, mast cell leukemia; SM-AHN, systemic mastocytosis with an associated hematologic neoplasm.

**Extended Data Fig. 8:**
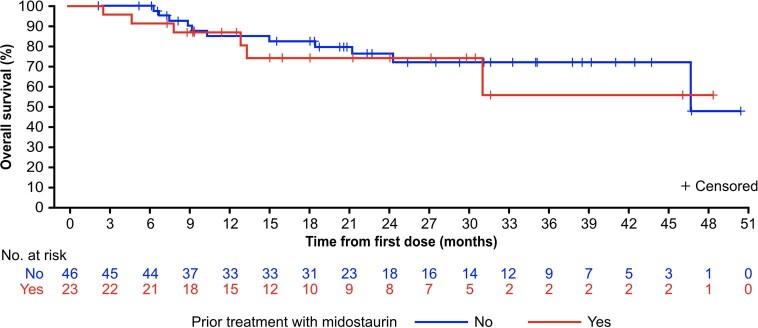
Overall Survival by Prior Midostaurin in the AdvSM Safety Population. AdvSM, advanced systemic mastocytosis.

**Extended Data Fig. 9:**
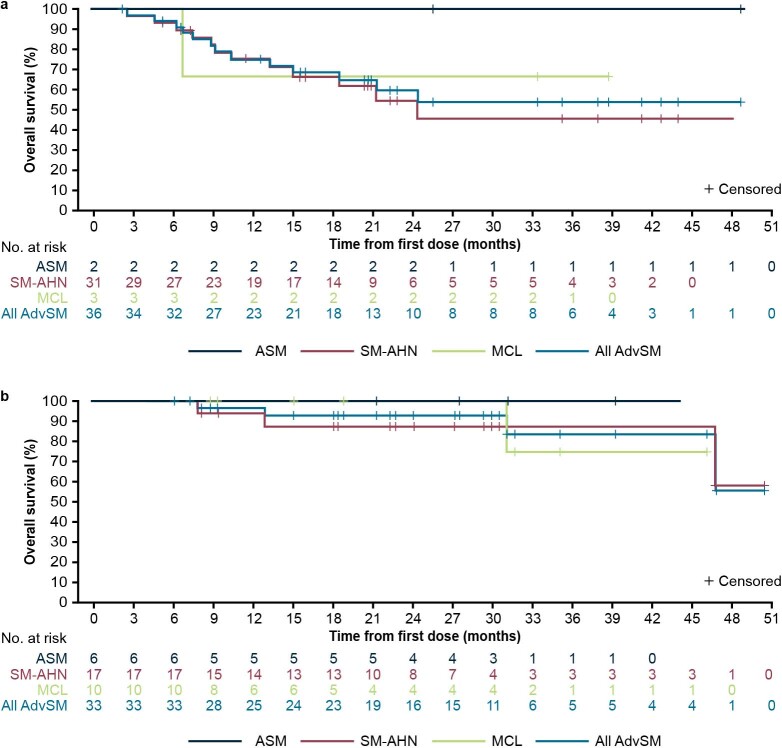
Overall Survival by *S/A/R* genotype in the AdvSM Safety Population. **a**, *S/A/R*-positive patients. **b**, *S/A/R*-negative patients. AdvSM, advanced systemic mastocytosis; ASM, aggressive systemic mastocytosis; MCL, mast cell leukemia; SM-AHN, systemic mastocytosis with an associated hematologic neoplasm; *S/A/R*, *SRSF2*, *ASXL1*, or *RUNX1*.

Estimated progression-free survival (PFS) rates in the response-evaluable population (*n* = 53) were 84% (95% CI, 73–94%) at 12 months and 63% (95% CI, 48–79%) at 24 months (Fig. 3a). During the study, six patients (9%) progressed to acute myeloid leukemia. Median OS was not reached (95% CI, 47–not estimable) in the overall AdvSM safety population (*n* = 69) with a median duration of follow-up of 23 months (Fig. 3b). Estimated 24-month OS rates were 76% (95% CI, 64–87%) overall, and 100%, 67% and 92% for patients with ASM, SM-AHN and MCL, respectively. OS was not substantially different for patients with or without prior midostaurin treatment (Extended Data Fig. 8); however, patients without *S/A/R* mutations had longer OS compared with those with *S/A/R* mutations (Extended Data Fig. 9). In a post hoc analysis, the mutation-adjusted risk score (MARS)^30^ was used for evaluation of PFS and OS risk in patients with AdvSM. MARS predicted both PFS (*P* = 0.0126) and OS (*P* = 0.0015), with a MARS ≥ 2 being associated with less favorable survival (OS shown in Supplementary Fig. 3). The OS in patients with a MARS of 0–1 was 100%.

### Pharmacokinetics

Steady-state mean plasma avapritinib concentration–time profiles are shown in Extended Data Fig. 1. Steady state was reached by day (D) 15. Steady-state plasma concentrations at doses of ≥200 mg QD exceeded the biochemical IC_90_ of 189 ng ml^−1^ for inhibition of KIT D816V, as measured by immunoblot in a xenograft model^24,31^, at most time points. Following single oral doses of 30 to 400 mg, the time to maximum plasma concentration (T_max_) ranged from 2 to 4 h. After single and repeat dosing of avapritinib, systemic exposure increased in a dose-dependent manner. The long mean plasma elimination half-life of avapritinib (range, 19.8 to 38.3 h) suggests that prolonged in vivo inhibition of KIT D816V, likely contributes to observed clinical activity, and supports QD dosing. The steady-state (cycle (C) 1, D15) geometric mean (percentage coefficient of variation; *n*), maximum plasma concentration (C_max_) and area under the plasma concentration–time curve (AUC_0-τ_) in patients at 200 mg QD was 433 ng ml^−1^ (62.2%; *n* = 18) and 7,340 h ng ml^−1^ (54.2%; *n* = 16), respectively.

### Patient-reported outcomes and symptom improvement

The AdvSM-symptom assessment form (AdvSM-SAF) patient diary evaluated symptoms across two domains—gastrointestinal and skin. Patients provided information about the severity of abdominal pain, nausea, vomiting, diarrhea, spots, itching, flushing and fatigue, and the frequency of vomiting and diarrhea. Reductions from baseline were seen in both the gastrointestinal and skin domains. Treatment with avapritinib also yielded consistent reductions in the patient-reported AdvSM-SAF total symptom score (TSS), which encompassed gastrointestinal and skin symptoms and fatigue. The mean TSS at baseline was 19.1 points (*n* = 40). Statistically significant improvements in symptoms occurred rapidly and were sustained through C11 (mean change from baseline of −10.9 points, *P* = 0.002, *n* = 20; Fig. 2e).

At baseline, approximately one-third of patients overall (29 patients; 34%) were using corticosteroids. After baseline, as a result of improvement in SM-associated symptoms, 19 patients (66%) reduced their corticosteroid usage, of whom 12 (41%) patients discontinued corticosteroid use entirely, while 7 (24%) were able to reduce the dose.

## Discussion

Patients with AdvSM have life-threatening organ damage and poor survival, caused by the increased proliferation of neoplastic mast cells driven by the *KIT* D816V mutation in ~95% of patients^6^. Midostaurin, until recently the only approved therapy for all subtypes of AdvSM, does not selectively target the KIT D816V mutant and only rarely do patients achieve a complete remission^7^. In a post hoc exploratory analysis of the pivotal midostaurin phase 2 study using IWG-MRT-ECNM criteria, responses occurred in 28% of patients with a 2% CR rate^32,33^.

Based on data from this phase 1 study and the ongoing phase 2 PATHFINDER (NCT03580655) study, avapritinib received United States FDA approval in June 2021 for adult patients with all subtypes of AdvSM, at a recommended dose of 200 mg orally QD. Due to the risks of ICB associated with severe thrombocytopenia, avapritinib is not recommended for the treatment of patients with AdvSM with a platelet count of <50 × 10^9^/l. The results of this phase 1 dose-escalation/expansion study demonstrated that potent and selective inhibition of KIT D816V by avapritinib elicited rapid, profound reductions of measures of mast cell disease burden, resolved organ damage, and improved patients’ symptoms across the spectrum of AdvSM subtypes.

Responses (CR/CRh/PR/CI) occurred in 75% of patients, including 36% achieving CR/CRh. These responses were adjudicated using stringent mIWG-MRT-ECNM response criteria, which require a sustained response with 12-week confirmation and full resolution of one or more C-findings. Responses were observed irrespective of disease subtype, prior therapy or presence of high-risk *S/A/R* mutations.

Notably, *KIT* D816V became undetectable in the BM of 30% of patients with AdvSM, using a highly sensitive ddPCR assay. This demonstrated that avapritinib induced deep molecular responses, an outcome that represents a new response benchmark in AdvSM. In an earlier multivariate analysis of 38 midostaurin-treated patients with AdvSM, a mere 25% reduction in *KIT* D816V RNA-expressed allele burden was statistically associated with prolonged survival^29^.

Consistent with these findings, early outcomes with avapritinib were excellent. While midostaurin and avapritinib have not been directly compared in a prospective, randomized trial, the survival of patients with AdvSM in the current study is noteworthy. The median OS was not reached in patients with AdvSM on avapritinib, with an estimated 24-month OS rate of 76% (95% CI, 64–87%). The published literature confirms the particularly poor prognosis of patients with MCL, with only a 24% probability of 24-month OS in this subgroup^34^. In the current study of avapritinib, the 24-month estimated median OS in patients with MCL was 92% (95% CI, 76–100%), while in the previous trial of midostaurin, 24-month OS in patients with MCL was 26% (95% CI, 6–54%)^7^. Importantly, avapritinib demonstrated high ORR rates and similar OS in patients with and without prior midostaurin exposure, suggesting that cross-resistance to avapritinib is rare.

Although patients with and without *S/A/R* mutations at baseline had similar response rates, the presence of *S/A/R* mutations or a MARS score of ≥2 was associated with shorter OS. These findings suggest that although avapritinib reduced mast cell burden and C-findings regardless of co-mutation status, patients with adverse myeloid co-mutations such as *S/A/R* were more likely to experience disease progression. In contrast, patients with AdvSM and a MARS score of 0–1 had excellent outcomes on avapritinib monotherapy in this study, as demonstrated by a 100% 24-month OS rate.

Avapritinib at ≥200 mg QD starting doses induced rapid responses, with a median time to CR/CRh/PR of 2 months, as compared with over 9 months for <200 mg QD starting doses. Given the poor overall survival characteristic of AdvSM, the rapid responses observed with a starting dose of at least 200 mg are clinically important.

Avapritinib was generally well tolerated with few discontinuations due to AEs. The most common AEs, including fluid retention, gastrointestinal effects and cytopenias, were consistent with those reported with other inhibitors of KIT/PDGFR, and usually responded to dose modification. Cytopenias were the most common ≥grade 3 AEs, and they occurred at a higher rate in patients who had baseline cytopenias. Cognitive events and ICB have previously been reported in patients with GIST treated with avapritinib^27^. Although symptoms such as brain fog and memory impairment are known disease features of SM^35^, cognitive effect AEs were less frequent in this AdvSM population than those previously observed in patients with GIST^27^, which may be related to the lower starting doses in this study.

ICB was more frequent in the AdvSM setting compared with GIST and was strongly associated with antecedent severe thrombocytopenia (platelets <50 × 10^9^/l). The incidence of ICB in AdvSM in the absence of severe thrombocytopenia (1%) was similar to the incidence in GIST. The observation of an association between severe thrombocytopenia and ICB led to the implementation of several patient selection and risk mitigation strategies in both the phase 1 EXPLORER and ongoing phase 2 PATHFINDER (NCT03580655) studies: exclusion of severely thrombocytopenic patients, increased platelet count monitoring, dose interruption in patients developing severe thrombocytopenia, and permanent treatment discontinuation after ICB of any grade. Antiplatelet agents and anticoagulants were avoided, and any coagulation abnormalities were corrected, if possible. The risk of ICB was mitigated in the aforementioned phase 2 study following the implementation of these measures. A proactive patient management strategy to minimize ICB risk will require platelet monitoring with dose interruption, reduction and platelet support in the event of severe thrombocytopenia.

The primary objectives of this dose-escalation/expansion study were to determine the MTD, safety and the RP2D of avapritinib, an investigational potent and selective KIT D816V inhibitor. Considering efficacy, tolerability and PK, the RP2D was identified as 200 mg QD based on consistent exposure above the KIT D816V in vivo IC_90_ and rapid time to response. At this dose, avapritinib induced deep and durable clinical, pathologic and molecular responses in patients with AdvSM, reduced disease-related symptoms, and was generally well tolerated.

The ongoing phase 2 PATHFINDER study has been designed to further characterize the safety and efficacy of avapritinib in patients with AdvSM at the 200 mg QD starting dose.

## Methods

### Patients

Patients aged ≥18 years with locally diagnosed AdvSM or relapsed or refractory myeloid malignancies with evidence of aberrant KIT or PDGFR signaling were enrolled in the dose-escalation phase (part 1), and only patients with locally diagnosed AdvSM according to WHO criteria^36^ were enrolled in the dose-expansion phase (part 2). Patients with ECOG performance status of 0–3 were eligible. Patients with platelets <25 × 10^9^/l were excluded initially; the threshold was subsequently raised to <50 × 10^9^/l to mitigate the risk of ICB. The complete inclusion and exclusion criteria are listed in Supplementary Table 3. Diagnoses were retrospectively adjudicated by central pathology and by an Adjudication Committee using WHO criteria. In patients with a centrally confirmed diagnosis of AdvSM, evaluable baseline C-findings and responses were defined using mIWG-MRT-ECNM^13^ consensus criteria (Supplementary Tables 4 and 5).

### Study objectives

The primary endpoints were MTD, RP2D and assessment of safety. Secondary endpoints included the ORR based on measurable C-finding responses per mIWG-MRT-ECNM criteria, which require 12-week confirmation of response. Responses in C-findings were centrally adjudicated by a Response Adjudication Committee (RAC) comprising a subset of study investigators (see below for further details and a list of RAC members). ORR included: CR or CRh (including complete resolution of all C-findings, elimination of marrow mast cell aggregates and serum tryptase <20 ng ml^−1^); PR (resolution of ≥1 C-finding and ≥50% reduction in marrow mast cells and serum tryptase); and clinical improvement (resolution of ≥1 C-finding).

Other secondary endpoints included PK, DOR and changes in measures of mast cell burden (percentage of BM mast cells, serum tryptase concentration, *KIT* D816V VAF by ddPCR (detection limit 0.17%), and spleen and liver volumes). PROs were measured by daily completion of AdvSM-SAF (described below) for patients in part 2 as a secondary outcome^37–39^. Exploratory endpoints included time to response, PFS and OS.

### Study treatment and design

This phase 1, open-label study comprised a dose-escalation phase (part 1) and a dose-expansion phase (part 2; Fig. 1). During dose escalation, patients received oral avapritinib at starting doses ranging from 30 to 400 mg QD in continuous 28-d treatment cycles. Dose escalation followed a 3 + 3 design (described below) until the MTD or RP2D was determined. During part 1, intra-patient dose escalation to assess dose levels not exceeding the MTD was allowed. Dose expansion was initially conducted at a starting dose of 300 mg QD; a second expansion cohort at 200 mg QD was subsequently introduced via protocol amendment. Dosing continued until patients experienced unacceptable toxicity, disease progression, death or withdrew consent.

#### 3 + 3 dose-escalation study design

The 3 + 3 dose-escalation design used cohorts of three to six patients. The first cohort received avapritinib at a starting dose of 30 mg daily. Dose escalation then proceeded at increments up to 100% until one or more patients treated at a given dose level had a ≥grade 2 non-hematologic AE possibly related to avapritinib or a grade 4 hematologic AE possibly related to avapritinib, or if the given dose exceeded the highest dose determined to be safe in the first-in-human study of avapritinib in patients with GIST (400 mg). Three patients were enrolled initially in each cohort with an additional accrual of three patients if the cohort required expansion due to DLT. Once the escalation cohort was full, up to three additional patients (diagnosis of AdvSM only) could be enrolled into an enrichment cohort at a lower dose. Data from these patients were intended for further exploration of PK, pharmacodynamics and safety in patients with AdvSM.

#### On-study corticosteroid treatment

Corticosteroid treatment, not exceeding doses administered during screening, was permitted; doses higher than 20 mg daily prednisone equivalent were avoided, and prolonged treatment (>14 d) with high-dose corticosteroids rendered patients ineligible for response assessment.

### Study oversight and review

The clinical trial registration number was NCT02561988 (date of preregistration, 15 September 2015). The study was designed by the sponsor (Blueprint Medicines) and study investigators. The full protocol was approved by the institutional review board (IRB) or independent ethics committee of each participating center: South Central–Berkshire Research Ethics Committee, Bristol; IRB, University of Pennsylvania; Dana-Farber Cancer Institute, Office for Human Research Studies; University of Utah IRB; University of Michigan Medical School IRB (IRBMED); The University of Texas MD Anderson Cancer Center IRB; Administrative Panels on Human Subjects in Medical Research, Stanford University (Stanford IRB); Western IRB (WIRB); and the Biomedical Research Alliance of New York (BRANY). The study was conducted in accordance with the Declaration of Helsinki, International Conference on Harmonization guidelines for Good Clinical Practice and local regulations. All patients provided written informed consent. Participants were not compensated, except for reimbursement of reasonable travel expenses. All authors had access to all data, reviewed and provided critical input to the manuscript and made the decision to submit for publication. All authors vouch for the validity of the study results and adherence to the protocol.

A formal independent data monitoring committee was not used for this study. The sponsor had access to the safety data on a regular basis, as this was an open-label study. The sponsor’s clinical study team hosted investigator teleconferences on a regular basis during the study. During part 1 of the study, the clinical study team and the investigators spoke by teleconference at the end of each treatment cohort to discuss and evaluate all of the gathered safety data. At the dose-escalation teleconference, safety information, including DLTs and all grade 2 or worse AEs reported during C1, and all available PK data were described and reviewed for each patient in the current dose cohort. Updated safety, PK and other data for all other ongoing patients, including data from later cycles, were also discussed. In addition, emerging safety and efficacy data were reviewed by the sponsor at quarterly safety meetings that included the study medical monitor, site personnel and biostatisticians.

In the event a patient was withdrawn from study drug administration or the follow-up phase of the study, the medical monitor was informed. If there was a medical reason for withdrawal, the patient remained under the supervision of the investigator or designee until the condition returned to baseline or stabilized.

Progress reports and notifications of serious unexpected adverse drug reactions were provided to the IRB/independent ethics committee and regulatory authority according to local regulations and guidelines.

#### Study investigators

Daniel J. DeAngelo: Department of Medical Oncology, Dana-Farber Cancer Institute, Boston, MA, USA; Michael W. Deininger: Versiti Blood Research Institute and Division Hematology and Oncology, Medical College of Wisconsin, Milwaukee, WI, USA; Jason Gotlib: Stanford Cancer Institute/Stanford University School of Medicine, Stanford, CA, USA; Prithviraj Bose: The University of Texas MD Anderson Cancer Center, Houston, TX, USA; Mark W. Drummond: Beatson Cancer Centre, Glasgow, UK; Elizabeth O. Hexner: Abramson Cancer Center, University of Pennsylvania, Philadelphia, PA, USA; Albert T. Quiery: University of Michigan, Ann Arbor, MI, USA; Deepti H. Radia: Guy’s & St Thomas’ NHS Foundation Trust, London, UK; William A. Robinson: University of Colorado, Denver, Aurora, CO, USA; Srdan Verstovsek: The University of Texas MD Anderson Cancer Center, Houston, TX, USA; Elliott F. Winton: Winship Cancer Institute, Emory University, Atlanta, GA, USA.

#### Central adjudication of diagnosis

To ensure a consistent diagnosis, retrospective central pathologic review of enrolled patients’ BM aspiration and biopsy samples and peripheral blood smears were assessed by an independent central pathologist. In addition, documentation (including site-entered data, as well as requested de-identified radiology reports and clinic notes) of baseline and historical (if relevant) C-findings were reviewed by the RAC to adjudicate and obtain consensus on SM diagnosis and subtyping based on the sum of available data. Diagnosis and subtyping were performed according to WHO criteria, with the following clarifications: the C-finding of weight loss was defined as documented weight loss of >10% within a 6-month (±3 months) period, and large osteolytic lesions were defined as ≥2 cm. These clarifications and strict requirement for documentation led to locally diagnosed ASM patients being reclassified as having indolent SM or smoldering SM by central diagnosis (based on data available to the RAC) for the purposes of data analysis. The MCL category also included MCL cases with evidence of AHN. The resulting comparison of local and centrally adjudicated diagnoses is presented in Supplementary Table 6.

#### Response adjudication committee members

Jason Gotlib (chair): Stanford Cancer Institute, Stanford University School of Medicine, Stanford, CA, USA; Daniel J. DeAngelo: Department of Medical Oncology, Dana-Farber Cancer Institute, Boston, MA, USA; Michael W. Deininger: Versiti Blood Research Institute and Division Hematology and Oncology, Medical College of Wisconsin, Milwaukee, WI, USA; Tracy I. George: ARUP Laboratories/University of Utah School of Medicine, Salt Lake City, UT, USA; Deepti H. Radia: Guy’s & St Thomas’ NHS Foundation Trust, London, UK.

### Analysis populations

Eligibility was determined locally during the 56-d screening period. All patients were followed for safety or toxicity (until 30 d after treatment discontinuation) and for survival. The frequency of assessments was per protocol schedule.

The safety population included all enrolled patients and was used to report AEs and PROs (in those who completed the questionnaire). The dose-determining population included all patients in a cohort in part 1 who received ≥21 prescribed avapritinib doses in C1 and completed follow-up through C1, or experienced DLT. The determination of the MTD and RP2D were based on the dose-determining population. The AdvSM safety population comprised all patients exposed to ≥1 dose of avapritinib, who had a diagnosis of AdvSM (ASM, SM-AHN or MCL) as centrally confirmed by the RAC. The AdvSM safety population was also used to report AEs and OS. The primary efficacy (response-evaluable) population included all patients with a centrally confirmed diagnosis of AdvSM (ASM, SM-AHN or MCL) who received ≥1 dose of avapritinib, and had at least one evaluable C-finding at baseline per mIWG-MRT-ECNM criteria as adjudicated by the RAC (MCL did not require baseline C-findings), and sufficient follow-up to be assessed for confirmed response (defined as being on study for at least six 28-d treatment cycles with ≥2 post-baseline BM assessments, or having an end-of-study assessment). The response-evaluable population was used to report adjudicated response rates and PFS. The PK population comprised all patients with sufficient plasma concentration–time data to reliably estimate the PK parameters of avapritinib.

The percentage change from baseline in spleen and liver volume as measured by central radiographic assessment using an MRI or a CT scan was summarized over time in the safety population. In addition, maximum percentage reduction was presented in waterfall plots.

Spleen response was determined for patients with baseline splenomegaly (spleen ≥ 5 cm by palpation) and was assessed by palpation or spleen volume by imaging (that is, ≥35% reduction from baseline in spleen volume based on central imaging); presence of either criterion was considered a response. DOR started at the first evidence of a response in any evaluable C-finding (per mIWG-MRT-ECNM criteria) and ended at the time a response was lost (that is, loss of response per mIWG-MRT-ECNM criteria). For patients who were still responding at the time of analysis, DOR was censored at the latest time point that the patient is assessed as having clinical improvement or better. OS was analyzed in the safety population and was defined as the time from first dose to the time of death due to any cause. PK data were summarized for patients with sufficient plasma concentration–time data. Descriptive statistics were used to summarize PK parameters for each dose level, as appropriate.

No imputation was performed for missing data elements. Where the date of onset of an AE was missing, event onset was assumed to be the date of treatment to conservatively report the event as treatment emergent.

### Study assessments

Responses were assessed according to the protocol schedule. Spleen size was assessed by palpation and by imaging with calculation of volume centrally. *KIT* D816V VAF in the BM (or in blood if unavailable) was centrally measured using a validated ddPCR assay with a lower limit of detection of 0.17% (Bio-Rad). Other somatic mutations in BM were centrally assayed at baseline by next-generation sequencing.

ORR was defined as the proportion of patients with a confirmed best response of CR, CRh, PR or clinical improvement by mIWG-MRT-ECNM consensus criteria. Time to CR/CRh/PR was defined as the time from the start of treatment to the time a CR/CRh/PR by mIWG-MRT-ECNM was first met. DOR was defined as the time from first documented response to the date of first documented progressive disease/loss of response or death due to any cause, whichever occurred first. OS was defined as the time from the start of treatment to the date of death. PFS was defined as the time from the start of treatment to the date of first documented progressive disease or death due to any cause, whichever occurred first. Progressive disease was defined per mIWG-MRT-ECNM criteria as worsening of ≥1 baseline C-finding confirmed for at least 4 weeks, or progression to acute myeloid leukemia.

#### Safety assessments

Safety assessments included determination of ECOG performance status, clinical laboratory testing, vital signs, electrocardiograms, brain imaging (MRI or CT scan) and physical examinations. AEs were graded according to National Cancer Institute Common Terminology Criteria (v4.03)^40^. Treatment-emergent AEs were defined as any AE occurring between the first dose of avapritinib through 30 d after the last dose of avapritinib, any event that was considered related to the study drug regardless of the start date of the event, or any event that was present at baseline but worsened intensity or was subsequently considered related to the study drug by the investigator.

#### Patient-reported outcomes assessment

The AdvSM-SAF comprises a ten-item questionnaire completed by the patient daily, specifically designed to assess symptoms related to AdvSM and measure the impact of treatments on patient symptom improvement^39^. It has been validated and demonstrated to be reliable and sensitive to clinical changes. The AdvSM-SAF assesses the severity of eight symptoms of AdvSM, including abdominal pain, nausea, vomiting, diarrhea, spots, itching, flushing and fatigue over a 24-h period on a numerical rating scale, and also measures the frequency of vomiting and diarrhea by asking patients to enter a discrete numerical value. Data were analyzed at the item level, domain level and total score level. Domain level analyses are:

- Gastrointestinal symptoms score (abdominal pain, nausea, vomiting and diarrhea severity (range 0–40))
- Skin symptom score (spots, itching and flushing severity (range 0–30))

The TSS was achieved by summing all eight severity items (range 0–80).

#### Pharmacokinetic assessments and analysis

On D1 and D15 of C1, blood samples for PK analysis were collected before the dose, and 0.5, 1, 2, 4, 8 and 24 h (C1D1 only) after the dose. C2D1 pre-dose concentration was used as the C1D15 24-h concentration for PK analysis. Additional pre-dose samples were collected on D1 of C3 and C4. Avapritinib PK parameters were obtained by non-compartmental analysis of the plasma concentration data versus nominal time using Phoenix WinNonlin© (version 8.0, Certara). Parameters estimated included C_max_, T_max_, AUC_0–‍24_; AUC_0–τ_, apparent oral clearance, apparent volume of distribution, terminal half-life, trough concentrations 24 h after dose and accumulation ratio.

Plasma concentration–time profiles across dose levels and dose versus systemic exposure (C_max_ and AUC) relationships were assessed graphically. Systemic exposure parameters of interest included the arithmetic and geometric mean, C_max_ and AUC, and 90% CIs.

### Additional statistical methods

The total number of patients enrolled in part 1 was dependent on the observed safety profile, which determined the number of patients per dose cohort, as well as the number of dose escalations required to identify the MTD and RP2D. It was expected that approximately 25 patients who met the criteria for the dose-determining population (described below) would be enrolled in part 1. In part 2, to adequately assess the safety of avapritinib, approximately 45 patients were planned for enrollment in cohort 1 at a starting dose of 300 mg QD, and approximately 10 patients were planned to be enrolled in cohort 2 at a starting dose of 200 mg QD. With 55 patients, there is approximately 94% probability of observing an AE that occurs at a frequency of ≥5%.

Primary ORR analysis was based on RAC-adjudicated responses in the response-evaluable population. ORR was estimated using frequency, percentage and two-sided 95% CIs based on the exact binomial distribution (Clopper–Pearson). Statistical testing on binomial proportion against a null of 28% was performed using one-sided *α* = 0.025. Wald test *P* value was presented. CR + CRh + PR rate was summarized similarly as ORR. Statistical testing on binomial proportion against a null of 17% was performed using one-sided *α* = 0.025.

OS, PFS and DOR were determined by Kaplan–Meier estimates, and 95% CIs were estimated using Greenwood’s formula^41^. Comparisons between two Kaplan–Meier functions was performed using a log-rank test.

Data analysis was performed using SAS version 9.3 software.

### Analysis data cutoff

The data cutoff for this analysis was 27 May 2020.

### Reporting Summary

Further information on research design is available in the Nature Research Reporting Summary linked to this article.

## Online content

Any methods, additional references, Nature Research reporting summaries, source data, extended data, supplementary information, acknowledgements, peer review information; details of author contributions and competing interests; and statements of data and code availability are available at 10.1038/s41591-021-01538-9.

## Supplementary information

Supplementary Information Supplementary Figs. 1–3 and Supplementary Tables 1–6

Reporting Summary

## Data Availability

Requests for the study protocol or the anonymized derived data from this study that underlie the results reported in this article will be made available, beginning 12 months and ending 5 years following this article publication, to investigators who sign a data access agreement and provide a methodologically sound proposal to medinfo@blueprintmedicines.com.
