# Supplementary material for: Safety and efficacy of avapritinib in advanced systemic mastocytosis: the phase 1 EXPLORER trial
Source: Nat Med. 2021 Dec 6;27(12):2183–91. doi: 10.1038/s41591-021-01538-9 (PMC8674134; doi:10.1038/s41591-021-01538-9)
Supplement: Supplementary file 1 — Supplementary Figs. 1–3 and Supplementary Tables 1–6 [file 41591_2021_1538_MOESM1_ESM.pdf]

nature portfolio

https://doi.org/10.1038/s41591-021-01538-9

Supplementary information

Safety and efficacy of avapritinib in advanced systemic mastocytosis: the phase 1 EXPLORER trial

In the format provided by the authors and unedited

Safety and efficacy of avapritinib in advanced systemic mastocytosis: the phase 1 EXPLORER trial

Supplementary Information

Table of Contents

Supplementary Figure 1. Adjudicated Responses per Modified IWG-MRT-ECNM Criteria, by Cycle, for Patients in the Response-Evaluable Population. 2

Supplementary Figure 2. Relationship between Depth of Clinical Response per mIWG-MRT-ECNM Criteria and Complete Molecular Response (Elimination of Measurable *KIT* D816V Variant Allele Fraction) in the Response-Evaluable Population. 3

Supplementary Figure 3. Overall Survival by Mutation-Adjusted Risk Score Category in the AdvSM Safety Population 4

Supplementary Table 1. Summary of Evaluable C-Findings per Modified IWG-MRT-ECNM Criteria Attributed to Systemic Mastocytosis at Baseline and Adjudicated Clinical Improvement (Response-Evaluable Population, *n* = 53). 5

Supplementary Table 2. Prior Anti-Neoplastic Therapies in the Response-Evaluable Population (*n* = 53). 6

Supplementary Table 3. Inclusion (a) and Exclusion (b) Criteria. 7

Supplementary Table 4. IWG-MRT-ECNM and Modified mIWG-MRT-ECNM Definitions of Evaluable C-Findings and Response Criteria. 11

Supplementary Table 5. IWG-MRT-ECNM and Modified (m)IWG-MRT-ECNM Criteria for Responses in Patients with AdvSM. 14

Supplementary Table 6. Diagnosis of AdvSM Subtype by Local Investigation Versus Central Response Adjudication Committee. 18

Supplementary Figure 1. Adjudicated Responses per Modified IWG-MRT-ECNM Criteria, by Cycle, for Patients in the Response-Evaluable Population.

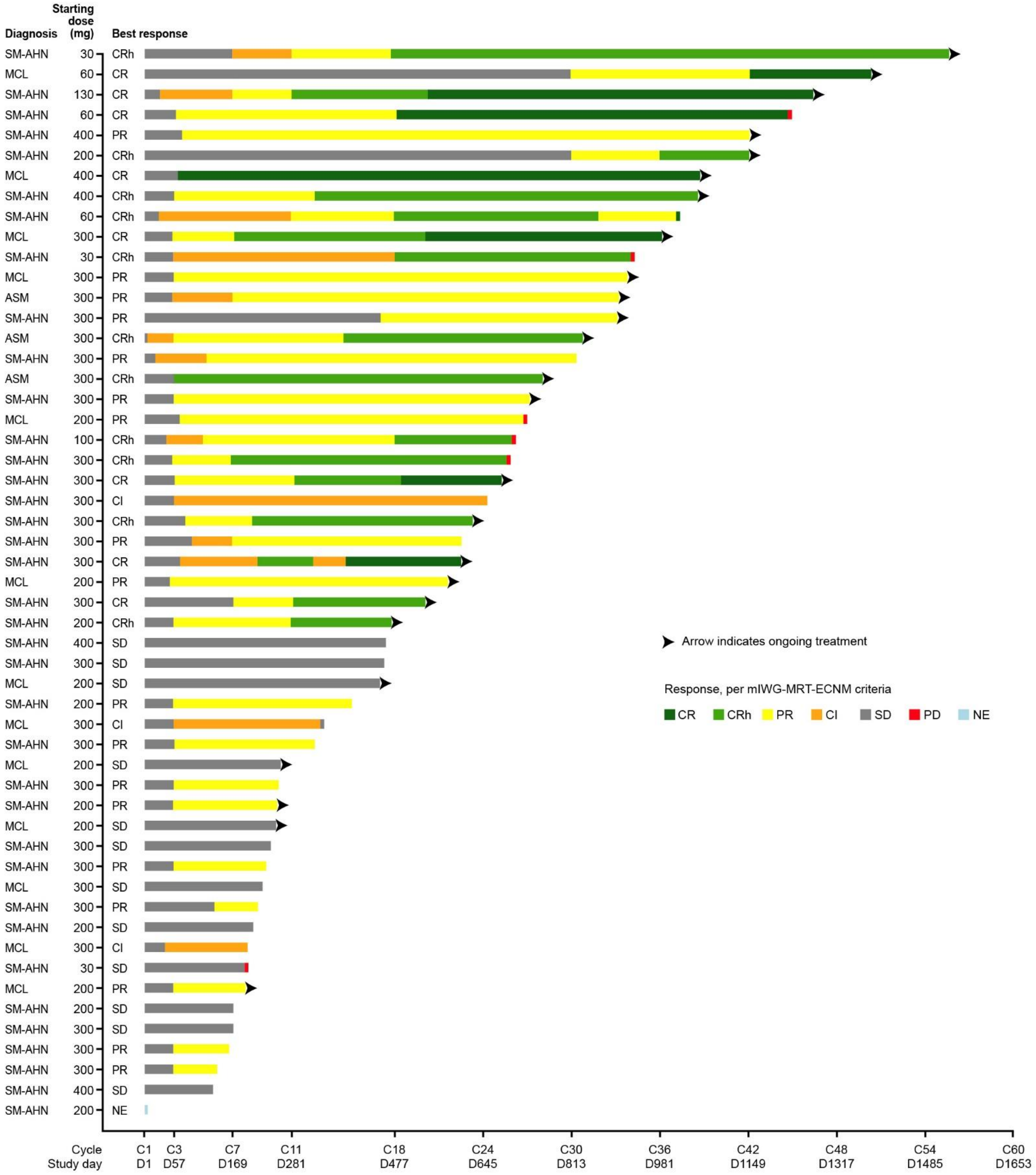

ASM, aggressive systemic mastocytosis; C, cycle; CI, clinical improvement; CR, complete remission; CRh, complete remission with partial recovery of peripheral blood counts; D, day; mIWG-MRT-ECNM, modified International Working Group-Myeloproliferative Neoplasms Research and Treatment and European Competence Network on Mastocytosis; MCL, mast cell leukemia; NE, not estimable; PD, progressive disease; PR, partial remission; SD, stable disease; SM-AHN, systemic mastocytosis with an associated hematologic neoplasm.

Supplementary Figure 2. Relationship between Depth of Clinical Response per mIWG-MRT-ECNM Criteria and Complete Molecular Response (Elimination of Measurable *KIT* D816V Variant Allele Fraction*) in the Response-Evaluable Population†.

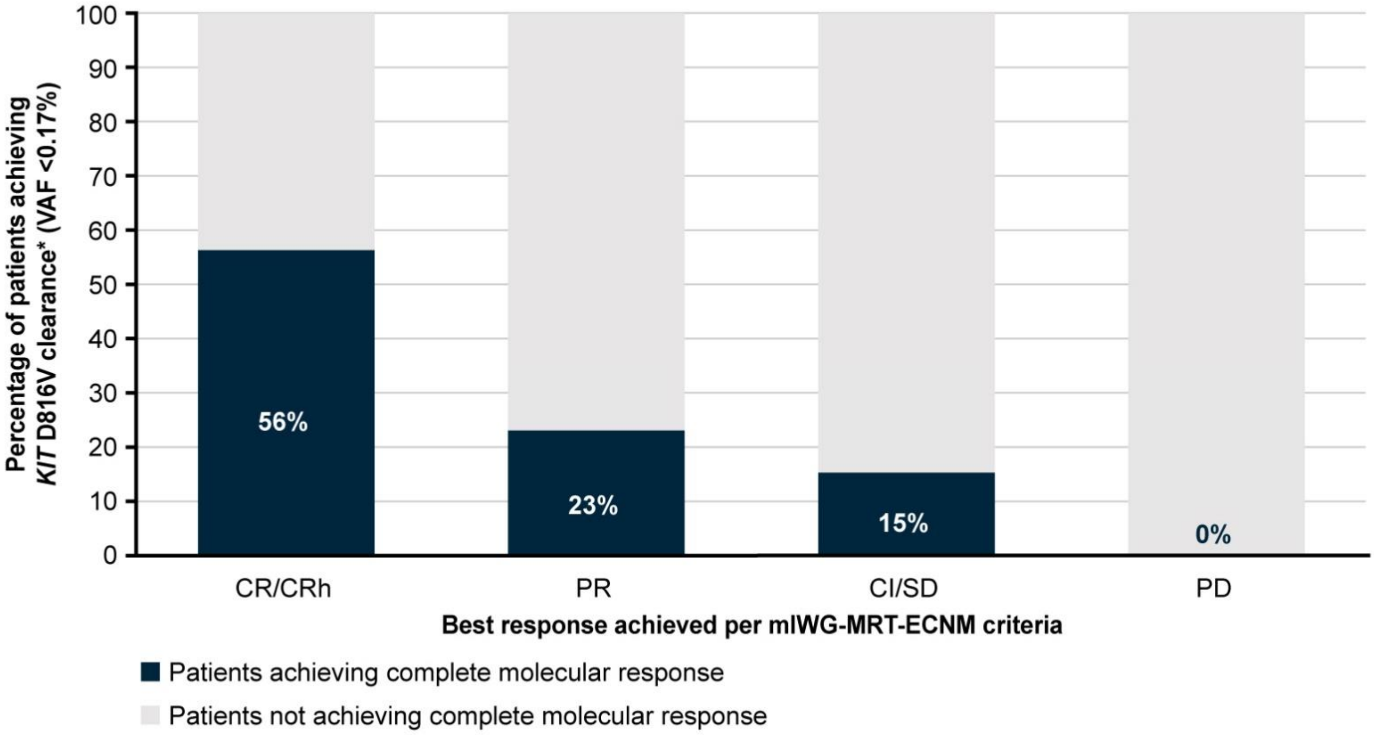

*Elimination of measurable *KIT* D186V VAF, with an assay lower limit of detection of 0.17%.

†Patients who had a baseline assessment of *KIT* D816V VAF in bone marrow are included in the analysis.

Minimum values of *KIT* D816V clearance at post-baseline were used for category grouping. Baseline was defined as the last observation before the first dose date of study drug, including pre-dose assessments on the first dose date. Percentages are based on the number of patients in each column.

CI, clinical improvement; CR complete remission; CRh, complete remission with partial recovery of peripheral blood counts; mIWG-MRT-ECNM, modified International Working Group-Myeloproliferative Neoplasms Research and Treatment and European Competence Network on Mastocytosis; PD, progressive disease; PR, partial remission; SD, stable disease; VAF, variant allele fraction.

Supplementary Figure 3. Overall Survival by Mutation-Adjusted Risk Score Category in the AdvSM Safety Population

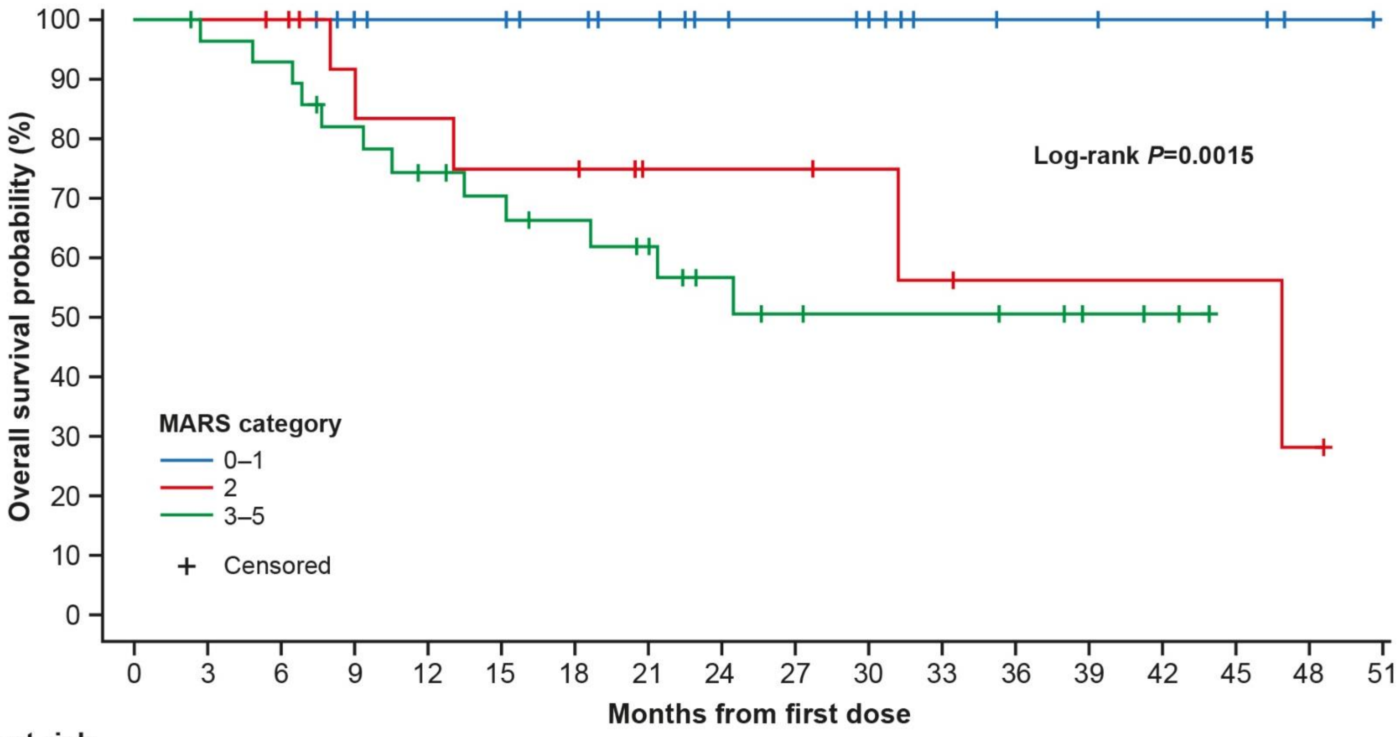

Patients at risk

**0–1**	25	25	25	22	19	19	17	15	12	11	9	5	4	4	3	3	1	0
**2**	15	15	14	11	10	9	9	5	5	5	4	3	2	2	2	2	1	0
**3–5**	29	27	26	22	19	17	15	12	9	7	6	6	5	3	2	0		

AdvSM, advanced systemic mastocytosis; MARS, mutation-adjusted risk score.

Supplementary Table 1. Summary of Evaluable C-Findings per Modified IWG-MRT-ECNM Criteria Attributed to Systemic Mastocytosis at Baseline and Adjudicated Clinical Improvement (Response-Evaluable Population, *n* = 53).

Presence of Evaluable C-Findings per Modified IWG-MRT-ECNM criteria* (n, %)	Patients with C-Finding at Baseline, n (%)	Patients with Clinical Improvement in C-finding, n (%)	Median Time to Clinical Improvement (months)
**Ascites**	10 (19)	4 (40)	7.4
**Pleural effusions**	2 (4)	2 (100)	1.9
**Liver function abnormalities**			
Direct bilirubin >1.5 × ULN	6 (11)	1 (17)	8.3
ALP >2.5 × ULN	18 (34)	12 (67)	2.9
AST >3.0 × ULN	0 (0)	–	–
ALT >3.0 × ULN	0 (0)	–	–
**Hypoalbuminemia**	7 (13)	4 (57)	3.3
**Splenomegaly ≥5 cm**	28 (53)	24 (86)	1.9
**Neutropenia**	3 (6)	0 (0)	–
**Anemia**			
Transfusion independent	15 (28)	3 (20)	3.7
Transfusion dependent	2 (4)	1 (50)	0.3
**Thrombocytopenia**			
Transfusion independent	18 (34)	4 (22)	3.1
Transfusion dependent	1 (2)	0 (0)	–

*Definitions of C-findings per mIWG-MRT-ECNM and criteria for measuring clinical improvement are presented in Supplementary Tables 4 and 5.

ALP, alkaline phosphatase; ALT, alanine aminotransferase; AST, aspartate aminotransferase; IWG-MRT-ECNM, International Working Group-Myeloproliferative Neoplasms Research and Treatment and European Competence Network on Mastocytosis; ULN, upper limit of normal.

Supplementary Table 2. Prior Anti-Neoplastic Therapies in the Response-Evaluable Population (*n* = 53).

Prior Treatment	Patients Receiving Prior Anti-Neoplastic Therapy, n (%)
**Any**	**32 (60)**
Midostaurin	17 (32)
Cladribine	7 (13)
Imatinib	3 (6)
Interferon	3 (6)
Hydroxycarbamide	3 (6)
Azacitidine	2 (4)
Brentuximab vedotin	2 (4)
Ibrutinib	2 (4)
Investigational antineoplastic drugs	2 (4)
Ruxolitinib	2 (4)
Chlorambucil	1 (2)
Decitabine	1 (2)
Obinutuzumab	1 (2)
Peginterferon alfa-2a	1 (2)
Rituximab	1 (2)

Table shows details of prior therapies received by patients that ended on or before the first dose date of avapritinib.

AdvSM, advanced systemic mastocytosis.

Supplementary Table 3. Inclusion (a) and Exclusion (b) Criteria.

a. Inclusion Criteria

1. Patients who are ≥18 years of age

Patients may be enrolled based on the local diagnosis. The diagnosis of AdvSM and the subclass will be confirmed by an independent pathologist retrospectively. Independent confirmation is not required for study entry.

2. For Part 1, patients must have 1 of the following diagnoses, based on WHO diagnostic criteria:
   - ASM
   - SM-AHN and the patient also has at least 1 C-finding attributable to SM. Laboratory abnormality C-findings should not be assessed until the required washout period from last cytoreductive therapy has been met. If a C-finding improves during the screening period, before dosing, and no longer meets criteria for evaluability, it can no longer be counted as a C-finding. The AHN must be myeloid, with the following exceptions that are excluded: AML, MDS that is very high- or high-risk as defined by the International Prognostic Scoring System for Myelodysplastic Syndromes (IPSS-R),[43] and Philadelphia chromosome-positive malignancies
   - MCL
   - Histologically or cytologically confirmed myeloid malignancy that was relapsed or refractory to standard treatments. AML, MDS that was very high- or high-risk as defined by the IPSS-R,[43] and Philadelphia chromosome-positive malignancies are excluded
   - On discussion with the sponsor, other relapsed or refractory hematologic neoplasms (e.g., evidence of aberrant KIT or PDGFR signaling) may be considered for enrollment
3. For Part 2, patients must have one of the following diagnoses, based on WHO diagnostic criteria:
   - ASM
   - SM-AHN. The AHN must be myeloid, with the following exceptions that are excluded: AML, MDS that is very high- or high-risk as defined by the IPSS-R,[43] and Philadelphia chromosome-positive malignancies
   - MCL
4. For Part 2, Cohort 2, patients must have at least 1 of the following measurable C-findings per modified IWG-MRT-ECNM criteria at baseline, attributed to SM, unless diagnosis is MCL, which does not require a C-finding.
   - Cytopenias:
     - ANC <1.0 × 10^9/L *or*
     - Hemoglobin <10 g/dL *or*
     - Platelet count <75 × 10^9/L

     NOTE: Cytopenias attributable to prior cytoreductive therapy or causes other than SM may not be used as C-findings
   - Symptomatic ascites or pleural effusion requiring medical intervention such as:
     - Use of diuretics (Grade 2) or
     - ≥2 therapeutic paracenteses or thoracenteses (Grade 3) at least 28 days apart over the 12 weeks before study entry and 1 of the procedures is performed during the 6 weeks before study start (C1D1).

- ≥Grade 2 abnormalities in direct bilirubin (>1.5 × upper limit of normal [ULN]), aspartate aminotransferase (AST; >3.0 × ULN), alanine aminotransferase (ALT; >3.0 × ULN), or alkaline phosphatase (>2.5 × ULN) with 1 of the following present:
    - Ascites *or*
    - Clinically relevant portal hypertension *or*
    - Liver MC infiltration that is biopsy-proven *or*
    - No other identified cause of abnormal liver function
- ≥Grade 2 hypoalbuminemia (<3.0 g/dL)
- A spleen that is palpable ≥5 cm below the left costal margin
- Transfusion-dependent anemia defined as:
    - Transfusion of ≥6 units packed red blood cells (PRBCs) in the 12 weeks before start of treatment (C1D1) *and*
    - Most recent transfusion occurring during the preceding 4 weeks *and*
    - Transfusion administered for hemoglobin ≤8.5 g/dL *and*
    - Reason for transfusion is not bleeding, hemolysis, or therapy-related

5. Patient has Eastern Cooperative Oncology Group (ECOG) performance status (PS) of 0–3
6. Patient or legal guardian, if permitted by local regulatory authorities, provides informed consent to participate in the study

**b. Exclusion Criteria**
1. Patient has any of the following within 14 days prior to the first dose of study drug:<br>• Alanine aminotransferase (ALT and aspartate aminotransferase (AST >3 × upper limit of normal (ULN; >5 × ULN if associated with clinically suspected liver infiltration by mastocytosis or another disease for which the patient enrolled into the study<br>• Total bilirubin >1.5 × ULN; >3 × ULN if associated with liver infiltration by the disease being treated or in the presence of Gilbert's Disease (in the case of Gilbert's disease, a direct bilirubin >2.0 ULN would be an exclusion)<br>• Estimated (Cockcroft-Gault formula) or measured creatinine clearance <40 mL/min<br>• Platelet count <50,000/μL (within 4 weeks of the first dose of study drug) or receiving platelet transfusion(s)<br>• Absolute neutrophil count (ANC) <0.5 × 10^9/L<br>2. Patient has eosinophilia and known positivity for the *FIP1L1-PGDFRA* fusion, unless the patient has demonstrated relapse or PD on prior imatinib therapy. Patients with eosinophilia (>1.5 × 10^9/L), who do not have a detectable *KIT* D816 mutation, must be tested for a *PDGFRA* fusion mutation by fluorescence in situ hybridization or PCR<br>3. Diagnosis of AML, MDS that is very high- or high-risk as defined by the IPSS-R, or a Philadelphia chromosome-positive malignancy<br>4. If the patient is receiving corticosteroids, and the dose has not been stable for ≥7 days. This exclusion criteria is not applicable if a patient has disease that is progressing and there is a safety concern around delaying the patient's study enrollment in order to stabilize the steroid dose<br>5. Patient received any antineoplastic therapy less than 14 days prior to the bone marrow assessment at screening, with the exception of hydroxyurea, which the patient may have received less than 7 days prior to the first dose of study drug<br>6. Patient received prior radiotherapy less than 14 days prior to the first dose of study drug<br>7. Patient requires therapy with a concomitant medication that is a strong inhibitor, strong inducer or moderate inducer of CYP3A4<br>8. Patient has had a major surgical procedure within 14 days of the first dose of study drug. Surgical procedures such as central venous catheter placement, BM biopsy and feeding tube placement are considered minor surgical procedures<br>9. History of another primary malignancy that has been diagnosed or required therapy within 1 year prior to the first dose of study drug. The following are exempt from the 1-year limit: completely resected basal cell and squamous cell skin cancer, curatively treated localized prostate cancer, GIST and completely resected carcinoma in situ of any site<br>10. QT interval corrected using Fridericia's formula (QTcF) >480 milliseconds<br>11. Patient has a history of prolonged QT syndrome or Torsades de pointes. Patient has a familial history of prolonged QT syndrome<br>12. Patient has a history of a seizure disorder (e.g. epilepsy) or requirement for antiseizure medication<br>13. Patient has a history of a cerebrovascular accident or transient ischemic attacks within 1 year prior to the first dose of study drug

14. Patient has a known risk of intracranial bleeding, such as a brain aneurysm or history of subdural or subarachnoid bleeding
15. A primary brain malignancy or metastases to the brain
16. Clinically significant, uncontrolled, cardiovascular disease, including congestive heart failure Grade III or IV according to the New York Heart Association classification, myocardial infarction or unstable angina within the previous 6 months, clinically significant, uncontrolled arrhythmias, or uncontrolled hypertension
17. Known diagnosis of human immunodeficiency virus infection or active viral hepatitis; viral testing is not required
18. Patient is unwilling or unable to comply with scheduled visits, drug administration plan, laboratory tests, or other study procedures and study restrictions
19. Female patients who are unwilling, if not postmenopausal or surgically sterile, to abstain from sexual intercourse or employ highly effective contraception from the first dose of study drug and for at least 6 weeks after the last dose of study drug. Men who are unwilling, if not surgically sterile, to abstain from sexual intercourse or employ highly effective contraception from the first dose of study drug and for at least 6 weeks after the last dose of study drug
20. Female patients who are pregnant, as documented by a serum beta human chorionic gonadotropin (β-hCG) pregnancy test consistent with pregnancy obtained within 7 days before the first dose of study drug. Women with β-hCG values that are within the range for pregnancy but are not pregnant (false-positives) may be enrolled with written approval of the Sponsor after pregnancy has been excluded. Women of nonchildbearing potential (i.e. women who are postmenopausal or have undergone hysterectomy; bilateral salpingectomy; or bilateral oophorectomy) do not require a serum β-hCG pregnancy test
21. Women who are breast feeding
22. Patient has a prior or ongoing clinically significant illness, medical condition, surgical history, physical finding, or laboratory abnormality that, in the Investigator's opinion, could affect the safety of the patient, alter the absorption, distribution, metabolism or excretion of the study drug, or impair the assessment of study results

Supplementary Table 4. IWG-MRT-ECNM and Modified mIWG-MRT-ECNM Definitions of Evaluable C-Findings and Response Criteria.

	IWG-MRT-ECNM Definition	IWG-MRT-ECNM Response Criteria	mIWG-MRT-ECNM Modifications
**Non-hematologic C-Findings**			
Ascites or pleural effusions	Symptomatic ascites or pleural effusion requiring medical intervention such as:<br>Use of diuretics (Grade 2) *or*<br>≥2 therapeutic paracenteses or thoracenteses (Grade 3) at least 28 days apart over 12 weeks before the start of treatment with one procedure performed 6 weeks before the start of treatment	Complete resolution of symptomatic ascites or pleural effusion (including trace or minimal on radiographic imaging) and no longer in need of diuretics for ≥12 weeks *and*<br>No longer in need of diuretics for ≥12 weeks *or*<br>No therapeutic paracenteses or thoracentesis for ≥12 weeks	As IWG-MRT-ECNM
Liver function abnormalities	≥Grade 2 abnormalities in direct bilirubin (>1.5 × ULN), AST (>3.0 × ULN), ALT (>3.0 × ULN), or ALP (>2.5 × ULN) in the presence of:<br>Ascites *and/or*<br>Clinically relevant portal hypertension, *and/or*<br>Liver MC infiltration that is biopsy-proven *or*<br>No other identified cause of abnormal liver function	Reversion of ≥1 LFTs to normal range for ≥12 weeks	As IWG-MRT-ECNM
Hypoalbuminemia	≥Grade 2 hypoalbuminemia (<3.0 g/dL)	Reversion of albumin to normal range for ≥12 weeks	As IWG-MRT-ECNM
Marked symptomatic splenomegaly	A spleen that is palpable **>5 cm** below the left costal margin and **patient endorses symptoms** of discomfort and/or early satiety	≥50% reduction in palpable splenomegaly (or ≥35% reduction in spleen volume based on 3D MRI or CT scan) and no endorsement of discomfort and/or early satiety for ≥12 weeks	Definition: Symptomatic or non-symptomatic splenomegaly palpable ≥5 cm below left costal margin. Response criteria: ≥35% reduction in spleen

			volume based on 3D MRI or CT scan for ≥12 weeks
Weight loss	N/A	N/A	Definition: Medically documented >10% weight loss in last 24 weeks (±12 weeks)<br>Response criteria: Reversion of >50% of weight loss in the 24 weeks preceding treatment
**Hematologic C-Findings**			
Neutropenia	≥Grade 3 ANC (<1.0 × 10^9/L)	≥100% increase *and* an absolute increase ≥0.5 × 10^9/L for ≥12 weeks	As IWG-MRT-ECNM
Anemia (transfusion-independent)	≥Grade 2 Hgb (<10 g/dL)	An increase in Hgb ≥2 g/dL that is maintained for ≥12 weeks	As IWG-MRT-ECNM
Anemia (transfusion-dependent)	Transfusion of ≥6 units PRBCs in the 12 weeks before the start of treatment *and*<br>Most recent transfusion occurring during the 4 weeks before the start of treatment *and*<br>Transfusions administered for Hgb ≤8.5 g/dL *and*<br>Reason for transfusions is not bleeding, hemolysis, or therapy-related	Transfusion independence for ≥12 weeks and maintenance of Hgb ≥8.5 g/dL at the end of the 12-week period of response duration	As IWG-MRT-ECNM
Thrombocytopenia (transfusion-independent)	≥Grade 2 thrombocytopenia (<75 × 10^9/L)	≥100% increase *and* an absolute increase ≥50 × 10^9/L and no need for platelet transfusion for ≥12 weeks	As IWG-MRT-ECNM
Thrombocytopenia (transfusion-dependent)	Transfusion of ≥6 units of apheresed platelets during 12 weeks preceding treatment *and*<br>≥2 units transfused during 4 weeks preceding treatment *and*<br>Transfusions administered for platelet count <20 × 10^9/L	Transfusion independence for ≥12 weeks *and* maintenance of platelet count ≥20 × 10^9/L	As IWG-MRT-ECNM

Grade is based on the Common Terminology Criteria for Adverse Events, Version 4.03.[31]

3D MRI, 3-dimensional magnetic resonance imaging; ALT, alanine aminotransferase; ALP, alkaline phosphatase; ANC, absolute neutrophil count; AST, aspartate aminotransferase; CT, computed tomography; Hgb, hemoglobin; IWG-MRT-ECNM, International Working Group-Myeloproliferative Neoplasms Research and Treatment and European Competence Network on Mastocytosis; LFT, liver function test; MC, mast cell; MRI, magnetic resonance imaging; N/A, not applicable; PBRC, packed red blood cell; ULN, upper limit of normal.

Supplementary Table 5. IWG-MRT-ECNM and Modified (m)IWG-MRT-ECNM Criteria for Responses in Patients with AdvSM.

Response	IWG-MRT-ECNM Criteria for Response[9]	mIWG-MRT-ECNM Modifications
Complete remission*	Requires all 4 of the following criteria, and response duration must be ≥12 weeks:<br>• No presence of compact neoplastic mast cell aggregates in the BM or other biopsied extracutaneous organ<br>• Serum tryptase level <20 ng/mL[†]<br>• Peripheral blood count remission defined as:<br>o ANC ≥1 × 10^9/L with normal differential (absence of neoplastic mast cells and blasts <1%) *and*<br>o Platelet count ≥100 × 10^9/L *and*<br>o Hgb level ≥11 g/dL<br>• Complete resolution of palpable hepatosplenomegaly and all biopsy-proven or suspected SM-related organ damage (C-findings)‡	As IWG-MRT-ECNM
CR with partial recovery of peripheral blood counts*	Not included	Requires all criteria for CR be met and response duration must be ≥12 weeks; however, patient may have residual cytopenias. The following minimum recovery of peripheral blood counts is required:<br>• ANC >0.5 × 10^9/L with normal differential (absence of neoplastic mast cells and blasts <1%) *and*<br>• Platelet count >50 × 10^9/L *and*<br>• Hgb level >8.0 g/dL
Partial remission*	Requires all 3 of the following criteria, and response duration must be ≥12 weeks, in the absence of both CR and PD:<br>• Reduction by ≥50% in neoplastic mast cells in the BM and/or other extracutaneous organ at biopsy demonstrating eligible SM-related organ damage<br>• Reduction of serum tryptase level by ≥50%[†]<br>• Resolution of ≥1 biopsy-proven or suspected SM-related organ damage (C-finding[s])[‡]	As IWG-MRT-ECNM

Clinical improvement*	Response duration must be ≥12 weeks<br>Requires 1 or more of the nonhematologic and/or hematologic response criteria to be fulfilled in the absence of CR, PR, or PD		As IWG-MRT-ECNM, plus absence of CR/CRh
Stable disease	Not meeting criteria for CR, PR, CI, or PD		Not meeting criteria for CR/CRh, PR, CI, or PD
Progressive disease§	*Requires at least one element from the criteria below; duration must be ≥8 weeks:*		*As IWG-MRT-ECNM; duration must be ≥4 weeks*
	**Baseline**	**Post baseline**	
	Any Grade 2 non-hematologic organ damage	Worsening by one grade *and*<br>Minimum 100% increase (doubling) of laboratory abnormality	Elements are per IWG-MRT-ECNM
	≥Grade 2 albumin	Worsening by one grade *and*<br>Decrease by ≥0.5 g/dL	
	≥Grade 3 non-hematologic organ damage	Minimum 100% increase (doubling) of laboratory abnormality	
	≥Grade 2 transfusion-independent anemia or thrombocytopenia	New transfusion dependence at 8 weeks of ≥4 units of RBCs or platelets	
	Transfusion-dependent anemia or thrombocytopenia	≥100% increase in the average transfusion frequency for an 8-week period compared with the 12 weeks preceding treatment	
	≥Grade 3 neutropenia	>50% decrease in neutrophil count *and*<br>Absolute decrease of neutrophil count of ≥0.25 × 10^9/L *and* Grade 4 (<0.5 × 10^9/L)	
	Baseline spleen size of not palpable or ≤5 cm	Development of at least 10 cm palpable symptomatic splenomegaly	Development of at least 10 cm palpable symptomatic splenomegaly *or*<br>Increase in spleen volume ≥25%

	Splenomegaly >5 cm	>50% worsening *and*<br>Development of ≥10 cm of palpable symptomatic splenomegaly compared with the baseline value	>50% worsening *and*<br>Development of ≥10 cm of palpable symptomatic splenomegaly compared with the baseline value *or*<br>Increase in spleen volume ≥25%
Loss of response	Loss of a documented CR, PR, or CI that must be for ≥8 weeks. Downgrading of CR to PR, or PR to CI is considered as such but is not considered a loss of response unless CI is also lost for ≥8 weeks. The baseline value for LOR is the pretreatment measurement(s) and not the nadir values during response.		As IWG-MRT-ECNM, plus loss or downgrading of CR/CRh

Guidelines for assessing response are as follows: (A) Only disease-related ≥Grade 2 organ damage is evaluable as a primary endpoint. (B) Response assessments of CR, PR, SD, PD, and loss of response should only be applied to these ≥Grade 2 organ damage findings in the context of trials. (C) Disease status at the time of patient removal from the study singularly relates to the updated status of initial ≥Grade 2 organ damage finding(s). (D) Exclusion of drug-related toxicity and/or other clinical issues (e.g., gastrointestinal tract bleeding in the case of worsening anemia/transfusion-dependence) should be undertaken before assigning the designation PD or loss of response in a patient with worsening of baseline ≥Grade 2 organ damage.

*Responses that are not maintained for a period of at least 12 weeks do not fulfil criteria for CR, PR, or CI; however, both maintained and unmaintained (<12 weeks duration) responses should be recorded each time they are observed in order to measure duration of response.

†Only valid as a response criterion if the pretreatment serum tryptase level is ≥40 ng/mL (i.e., if pretreatment serum tryptase is <40 ng/mL, it will not be considered as a criterion in evaluation of response).

‡Biopsy of organ(s) in addition to the bone marrow to evaluate for SM-related organ damage may be considered.

§Preservation of at least one CI finding permits a patient to maintain the response of CI if one or more CI findings are lost but none meet criteria for PD. However, if one or more of the CI findings become PD, then the CI finding assignment is lost and the patient meets criteria for PD. The baseline value for evaluating PD is the pretreatment measurement(s). The PD findings must be considered related to the underlying disease and not to other clinical factors. Progression of an underlying chronic myeloid neoplasm to acute myeloid leukemia is also considered PD.

AdvSM, advanced systemic mastocytosis; ANC, absolute neutrophil count; BM, bone marrow; CI, clinical improvement; CR, complete remission; CRh, complete remission with partial recovery of peripheral blood counts; Hgb, hemoglobin; IWG-MRT-ECNM, International Working Group-Myeloproliferative Neoplasms Research

and Treatment and European Competence Network on Mastocytosis; LOR, loss of response; PD, progressive disease; PR, partial remission; RAC, Response Adjudication Committee; RBCs, red blood cells; SD, stable disease; SM, systemic mastocytosis.

Supplementary Table 6. Diagnosis of AdvSM Subtype by Local Investigation Versus Central Response Adjudication Committee.

Local Investigator Diagnosis, n	**Centrally Adjudicated Diagnosis, n**						
	**ASM**	**SM-AHN**	**MCL**	**ISM**	**SSM**	**Not SM**	**Total**
ASM	7	15	1	13	1	0	37
SM-AHN	1	33	1	0	0	1	36
MCL	0	0	11	0	0	0	11
SSM	0	0	0	1	1	0	2
**Total**	**8**	**48**	**13**	**14**	**2**	**1**	**86**

The grey boxes indicate numbers of patients in whom central adjudication did not result in a change from the local diagnosis.

ASM, aggressive systemic mastocytosis; ISM, indolent systemic mastocytosis; MCL, mast cell leukemia; SM, systemic mastocytosis; SM-AHN, systemic mastocytosis with an associated hematologic neoplasm; SSM, smoldering systemic mastocytosis.
